# Ibotenic Acid and Muscimol in *Amanita muscaria*: Chemistry, Sources of Variability, Analytical Determination, and Toxicological Significance

**DOI:** 10.3390/molecules31183232

**Published:** 2026-09-13

**Authors:** Andrzej Günther, Barbara Bednarczyk-Cwynar, Michał Tomczyk

**Affiliations:** 1 Department of Organic Chemistry, Faculty of Pharmacy, Poznan University of Medical Sciences, Collegium Pharmaceuticum 2 (CP.2), ul. Rokietnicka 3, 60-806 Poznań, Poland; 2 Center of Innovative Pharmaceutical Technology (CITF), ul. Rokietnicka 3, 60-806 Poznań, Poland; 3 Department of Biology and Pharmacognosy, Faculty of Pharmacy with the Division of Laboratory Medicine, Medical University of Białystok, ul. Mickiewicza 2a, 15-222 Białystok, Poland; michal.tomczyk@umb.edu.pl

**Keywords:** *Amanita muscaria*, ibotenic acid, muscimol, isoxazole metabolites, chemical variability, post-harvest transformation, LC–MS/MS, analytical toxicology, fungal natural products

## Abstract

*Amanita muscaria* contains two structurally related neuroactive isoxazole metabolites, ibotenic acid (IA) and muscimol (MUS), which differ markedly in pharmacological activity. IA acts as an agonist at NMDA and metabotropic glutamate receptors, whereas MUS is a potent GABAergic agonist. This review critically evaluates their chemistry and biosynthesis, biological variability, post-harvest transformation, analytical determination, and toxicological significance. Direct evidence demonstrates interspecific variation within *Amanita* section *Amanita*. Developmental evidence for *A. muscaria* is limited but direct: an older maturation study identified tissue-dependent changes in IA and MUS, while concentrations calculated for the whole fruiting body remained comparatively stable; observations in *A. subglobosa* provide additional species-specific evidence. Controlled studies also demonstrate processing-related changes during drying and heating, whereas storage effects remain condition-dependent and less completely characterized. Cross-study comparison is constrained by differences in species identification, tissue selection, developmental stage, hydration, sample handling, normalization, and analytical procedure. Quantitative study-level comparison shows that LC–MS/MS and UHPLC–MS/MS, capillary electrophoresis, GC–MS, NMR, and rapid ambient-MS approaches serve different matrices and analytical purposes. Their reported sensitivity, recovery, precision, and calibration performance cannot be ranked directly across studies, and matrix-specific validation remains essential. Broader fungal research provides mechanistic context for possible environmental regulation, but direct controlled evidence for stress-dependent IA biosynthesis in *A. muscaria* remains sparse. The IA/MUS ratio is best treated as a secondary chemical descriptor alongside absolute concentrations and a clearly defined normalization basis. Toxicological interpretation additionally requires consideration of total dose, preparation, matrix composition, and individual susceptibility.

## 1. Introduction

*Amanita muscaria* (L.) Lam. is one of the most recognizable toxic mushrooms and a well-known source of neuroactive isoxazole metabolites. Its principal toxicologically relevant constituents are ibotenic acid (IA) and muscimol (MUS), whereas muscarine, despite its historical association with the species, occurs at comparatively low concentrations and does not account for the characteristic central nervous system manifestations of fly agaric poisoning [1,2,3,4]. Ingestion of *A. muscaria* or related species may produce the pantherina–muscaria syndrome, which includes gastrointestinal symptoms, confusion, agitation, hallucinations, ataxia, somnolence, and, less commonly, severe central nervous system depression [2,3,5].

Ibotenic acid and muscimol are structurally related but pharmacologically distinct. Ibotenic acid is an amino acid-like isoxazole compound that acts as a glutamate-receptor agonist, whereas muscimol is a potent agonist of ionotropic GABA receptors [1,6,7]. Ibotenic acid can undergo decarboxylation to muscimol, making their relative abundance sensitive to chemical transformation. Fresh tissue, dried material, cooked preparations, extracts, and stored samples may therefore differ in both absolute concentrations and relative proportions of the two compounds. The ibotenic acid-to-muscimol concentration ratio (IA/MUS ratio) may provide a useful description of the chemical profile of a sample, although it should not be interpreted as an independent predictor of clinical outcome. Total dose, matrix composition, preparation method, absorption, co-exposures, and individual susceptibility also contribute to toxicological variability.

Available evidence points to taxonomic and developmental variability, although the number of independent datasets is small. In the largest recent comparative study, Su et al. detected ibotenic acid and muscimol in 10 of 24 species examined within *Amanita* section *Amanita*. Developmental data are more limited. Su et al. reported lower concentrations in mature specimens of *A. subglobosa* [8], whereas an earlier study followed *A. muscaria* through maturation and found tissue-dependent changes in IA and MUS, with comparatively stable concentrations when the whole fruiting body was considered [9]. These studies establish that species identity, developmental stage, and tissue selection can affect the measured chemical profile, but they do not define a universal pattern for *A. muscaria* populations. Geographic origin, hydration, storage history, and analytical procedure remain additional sources of variation.

Importantly, concentration changes within individual anatomical compartments should not be equated with changes in total toxin burden, particularly during fruiting-body expansion and changes in tissue water content.

Post-harvest handling is supported by direct experimental evidence as an important source of chemical variation. Drying and heating may promote decarboxylation of ibotenic acid, while cooking can combine transformation with leaching into water. Storage effects are condition-dependent. IA and MUS were stable under specific dry/cool/dark and salted/refrigerated conditions tested experimentally, whereas stability across other storage regimes remains insufficiently characterized [10,11].

Extraction conditions, including solvent composition, pH, temperature, and duration, can alter analyte recovery and generate preparation-specific profiles [12,13,14,15]. Consequently, concentrations obtained from fresh mushrooms, dried powders, hydroalcoholic extracts, biological fluids, and commercial formulations are not directly comparable without detailed information on sample handling and method validation.

Reliable interpretation therefore depends on appropriate analytical methodology. Ibotenic acid and muscimol are small, highly polar molecules that may be poorly retained in conventional reversed-phase chromatographic systems. Hydrophilic interaction liquid chromatography coupled with tandem mass spectrometry, LC–MS/MS, and UHPLC–MS/MS provide high selectivity and sensitivity and have been applied to mushroom material and biological specimens [8,12,16,17].

Capillary electrophoresis and CE–ESI-MS/MS offer complementary approaches, particularly for charged analytes and small sample volumes, but their performance depends strongly on control of migration conditions, matrix effects, and reproducibility [13,14]. Differences in extraction, calibration, internal standards, detection limits, reporting units, and sample normalization remain major obstacles to cross-study comparison.

The biosynthetic basis of ibotenic acid production has become clearer following the identification of glutamate hydroxylation as the initial step in its pathway [18]. This finding links ibotenic acid formation to a defined enzymatic process and provides a mechanistic foundation for future studies of pathway regulation. However, direct evidence that temperature, drought, soil chemistry, nitrogen availability, heavy metals, microbial competition, or host-tree stress regulate ibotenic acid biosynthesis in *A. muscaria* remains limited. The general literature on fungal secondary metabolism shows that environmental and developmental signals can modify metabolite production [19,20,21,22,23,24,25,26], but such findings constitute indirect support rather than proof of stress-dependent regulation of the ibotenic acid pathway. Environmental modulation consequently remains a testable hypothesis rather than an established feature of the ibotenic acid pathway.

Interest in the chemistry of *A. muscaria* has increased with the availability of commercial extracts, gummies, and other products marketed as legal psychoactive or nootropic preparations. Their composition may reflect uncontrolled variation in raw material, developmental stage, drying, extraction, formulation, storage, or adulteration. Recent public health and regulatory reports have raised concerns about inconsistent labelling, undeclared psychoactive substances, uncertain dosing, and adverse clinical outcomes [27,28,29,30]. These products create additional analytical and regulatory challenges because their chemical profiles may differ substantially from those of fresh fruiting bodies.

Several reviews have already approached *Amanita muscaria* from complementary perspectives. Michelot and Melendez-Howell [4] provided a broad synthesis of its chemistry, biology, toxicology, and ethnomycology, whereas Stebelska [6] reviewed analytical methods and the biological activity of fungal hallucinogens, including IA and MUS. Voynova et al. [1] placed greater emphasis on the toxicological, pharmacological, and potential biomedical relevance of *A. muscaria*. More recently, Ordak [31] examined the mushroom in the context of emerging psychoactive-substance use, with particular attention to clinical toxicity, patterns of consumption, and public health concerns. These reviews overlap with several topics considered here, especially pharmacology and toxicology. The contribution of the present review is therefore not simply broader coverage of *A. muscaria*, but a different treatment of the underlying evidence.

Here, the emphasis is on how chemical findings can be compared and interpreted across studies. Evidence obtained directly from *A. muscaria* is separated from observations in related species and from mechanistic inference; biological variation is considered separately from changes introduced by post-harvest processing and analytical procedures; and analytical methods are compared using study-level performance data rather than platform descriptions alone. The review also brings these elements together in a reporting framework intended to make future IA and MUS datasets more comparable. This approach is designed to show not only what has been reported, but also how securely individual conclusions are supported and where methodological differences prevent apparently similar results from being combined. The scope of the principal earlier reviews is compared with the present synthesis in Supplementary Table S1.

Throughout this review, *A. muscaria* remains the focal species. Evidence from other members of *Amanita* section *Amanita* is used to define taxonomic context or to address questions for which species-specific data are limited, and the source species is stated explicitly. Findings from related *Amanita* species are not treated as direct evidence for *A. muscaria* unless the study itself included authenticated *A. muscaria* material. Evidence from non-Amanita fungal systems is used only as mechanistic context, whereas commercial products of uncertain fungal identity are treated as product matrices rather than as authenticated *A. muscaria* material.

## 2. Literature Search and Evidence Assessment

This review was conducted as a critical narrative synthesis of evidence from natural product chemistry, fungal biosynthesis, analytical toxicology, neuropharmacology, clinical toxicology, and public health. The SANRA domains were used as a quality-appraisal framework for the narrative synthesis, particularly with respect to the formulation of the review aims, description of the literature search, referencing of key statements, and presentation of the strength of the underlying evidence [32]. SANRA was not used as a literature-search methodology. Literature identification and selection were conducted separately using the database-specific search procedures described below and documented in Supplementary Table S2. Literature searches were performed in PubMed, Scopus, Web of Science, and Google Scholar. CrossRef, publisher databases, and the reference lists of relevant publications were used to identify additional records. The final update search was completed on 10 August 2026.

Search terms combined “*Amanita muscaria*”, “fly agaric”, “ibotenic acid”, “muscimol”, “muscazone”, and “muscarine” with terms related to biosynthesis, chemical variability, developmental stage, tissue distribution, drying, heating, storage, extraction, LC–MS/MS, UHPLC–MS/MS, UPLC–MS/MS, capillary electrophoresis, poisoning, and commercial mushroom products. The broader literature on fungal secondary metabolism and environmental stress was included only when it provided mechanistic context relevant to the regulation of fungal metabolites.

Studies were considered eligible when they addressed at least one of the following areas: identification or quantification of ibotenic acid and muscimol; biosynthesis or chemical transformation; taxonomic, developmental, tissue-specific, or processing-related variability; analytical methodology; pharmacological or toxicological effects; clinical or veterinary exposure; or the composition and safety of commercial products. Priority was given to peer-reviewed studies with clearly identified biological material, quantitative analytical data, validated methods, or direct toxicological relevance. Regulatory and public health documents were included when they contained recent exposure or product-safety information not available in the peer-reviewed literature.

Evidence was appraised at the level of the specific claim for which a study was cited, rather than assigning a single label to an entire publication. Three categories were used. Direct evidence refers to measurements or experiments that address the claim in the relevant Amanita material, chemical system, analytical matrix, or documented exposure. Indirect evidence includes observations that are experimentally relevant but require extrapolation—for example, results from another *Amanita* species, a broader metabolomic analysis that did not quantify IA or MUS specifically, or mechanistic findings from other fungal systems. Hypothesis-generating evidence denotes biologically plausible relationships for which no direct test of the proposed IA/MUS effect is yet available.

This claim-level approach is important because the same publication may occupy different evidence categories depending on how it is used. A study may, for example, provide direct evidence of general chemical differences between fungal tissues while offering only indirect support for a tissue-specific distribution of IA and MUS. The classification assigned to each primary study and claim is reported in Supplementary Table S3.

Supplementary Table S3 records the species or matrix, sample size where available, analytical approach, principal finding, main limitation, evidence category, and section of the review in which each study contributes to the synthesis. Reviews and general background sources are identified separately from primary evidence and were not counted as independent experimental datasets.

Taxonomic scope was recorded separately from evidential strength. Thus, a measurement made directly in *A. subglobosa* constitutes direct evidence for that species but becomes indirect evidence when used to support a species-specific claim about *A. muscaria*. The same distinction applies to other members of *Amanita* section *Amanita* and to studies in which fungal identity was not independently established.

For biological studies, the appraisal considered species authentication, sample size, developmental stage, tissue selection, geographic origin, sample storage, processing conditions, concentration units, and normalization to fresh or dry weight. Analytical studies were additionally evaluated for extraction procedure, calibration, internal standards, detection and quantification limits, recovery, precision, reproducibility, and matrix effects. Clinical reports were assessed with respect to exposure verification, analytical confirmation, co-exposures, patient characteristics, and the completeness of outcome reporting.

Because the available evidence is heterogeneous in study design, biological material, analytical platform, and outcome reporting, no quantitative meta-analysis was attempted. The synthesis instead focuses on the consistency, limitations, and interpretative strength of the available evidence.

## 3. Chemistry and Biosynthesis of Ibotenic Acid and Muscimol

### 3.1. Chemical Properties and Structural Relationship

Ibotenic acid and muscimol are small, strongly ionizable isoxazole metabolites whose chemical behaviour is not fully represented by the neutral structural formulas commonly used in reaction schemes. Ibotenic acid contains an amino group, a carboxyl group, and a 3-hydroxyisoxazole moiety. X-ray crystallography has established a zwitterionic monohydrate in the crystalline state [33]. Its several ionizable sites also make the charge distribution pH-dependent in solution. Ibotenic acid contains a stereogenic α-carbon. The natural product has been described as racemic in the chemical literature [34,35]. Analytical methods that do not resolve enantiomers therefore report total IA without defining its enantiomeric composition.

Muscimol contains no stereogenic centre and is achiral. It likewise cannot be described adequately by a single neutral structural formula. The 3-hydroxyisoxazole system allows alternative protonation and tautomeric representations, while protonation of the aminomethyl group gives rise to strongly polar ionic and zwitterionic forms. The zwitterionic structure of muscimol has been characterized crystallographically [36], and classical structure–activity work reported pKa values of approximately 4.8 and 8.4 [37]. These acid–base properties are relevant not only to its relationship with GABA but also to its behaviour during extraction and separation.

The pronounced polarity of IA and MUS follows in part from these ionization characteristics. Their retention in conventional reversed-phase systems can therefore be weak and sensitive to mobile-phase pH, whereas HILIC, electrophoretic separation, ion-interaction approaches, or derivatization may provide more suitable alternatives for particular matrices [12,13,14,16,17]. Mobile-phase conditions can also influence electrospray response and apparent recovery, making pH control an analytical variable rather than a purely chromatographic detail. The structural differences between IA and MUS are also reflected in their receptor pharmacology: IA acts as an agonist at N-methyl-D-aspartate (NMDA) receptors and at group I and II metabotropic glutamate receptors, whereas MUS primarily activates ionotropic GABA receptors [6,7,38,39].

**Figure 1:**
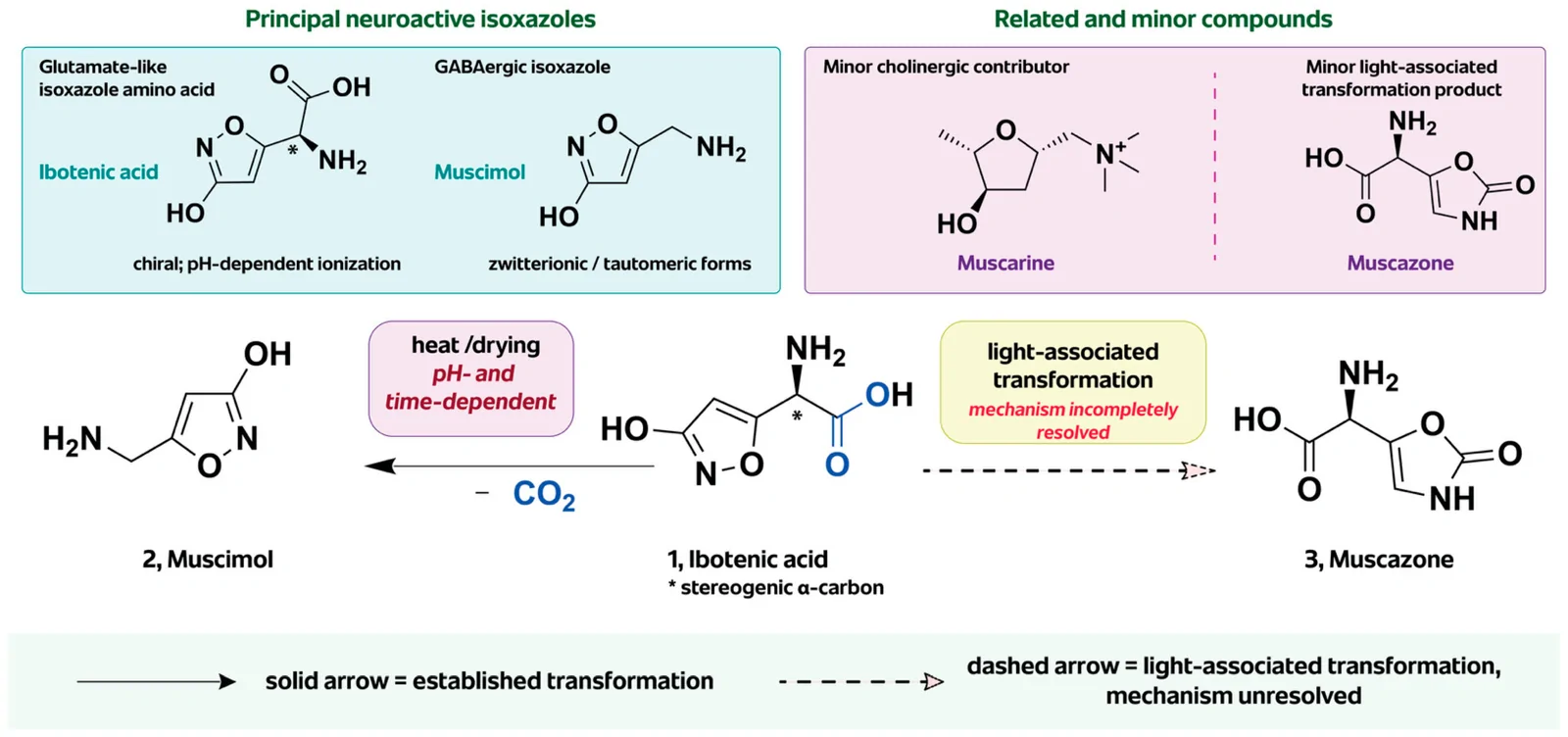
Chemical structures and principal transformations of ibotenic acid, muscimol, and related compounds. Ibotenic acid contains a stereogenic α-carbon, whereas muscimol is achiral; both ibotenic acid and muscimol display pH-dependent ionization; zwitterionic forms of both compounds have been characterized structurally [33,36,37]. The solid arrow from ibotenic acid to muscimol represents decarboxylation with release of CO_2_ under defined processing conditions [10,40]. The dashed arrow toward muscazone denotes a light-associated transformation whose detailed mechanism remains incompletely resolved [41,42]. Muscarine is chemically distinct from the principal isoxazole system [1,4].

The chemical structures of ibotenic acid, muscimol, muscarine, and muscazone, together with the principal transformations of ibotenic acid, are shown in Figure 1.

The two compounds are connected through decarboxylation:

**ibotenic acid → muscimol + CO_2_**

This transformation is central to the chemistry of *A. muscaria* because it changes both molecular structure and pharmacological activity. However, the extent of conversion depends on temperature, time, moisture, pH, matrix composition, and sample handling. Statements implying complete or uniform conversion during drying or heating are therefore not supported without process-specific analytical data [10,40].

Muscazone has long been associated with the photochemical transformation of ibotenic acid [1,41,42]. The early studies established the compound and its relationship to *A. muscaria* chemistry, but they do not justify treating its formation as a single, mechanistically resolved pathway of simple “UV photodegradation”. The available evidence does not clearly distinguish degradation from photochemical rearrangement or isomerization under different conditions. Muscazone is therefore better described as a minor light-associated transformation product of ibotenic acid. Its quantitative formation, competing pathways, and dependence on irradiation conditions remain insufficiently characterized by modern kinetic or mass-balance studies.

### 3.2. Biosynthesis of Ibotenic Acid and the Ibo Gene Cluster

The genetic basis of ibotenic acid biosynthesis was substantially clarified by Obermaier and Müller, who identified a seven-gene biosynthetic gene cluster in *A. muscaria* comprising *iboA*, *iboF*, *iboD*, *iboC*, *iboG1*, *iboH*, and *iboG2* [18]. The most firmly established enzymatic step is catalysed by IboH, an Fe(II)/2-oxoglutarate-dependent dioxygenase. Recombinant IboH converted *L*-glutamate to 3-hydroxy-L-glutamate, whereas glutamine was not accepted as the corresponding initial substrate under the experimental conditions. This reaction establishes L-glutamate hydroxylation as the first committed step of the pathway [18].

The functions assigned to the remaining cluster proteins are less firmly established. Sequence-based analysis led to the proposal that IboA, an adenylating enzyme, activates the terminal carboxyl function during amide formation, whereas the flavin-dependent monooxygenase IboF participates in formation of the N–O bond. The paralogous PLP-dependent proteins IboG1 and IboG2 were proposed to participate in substitution and cyclization reactions leading toward the isoxazole framework. Tricholomic acid was suggested as the most likely first cyclic intermediate, with the cytochrome P450 enzyme IboC proposed to catalyse its desaturation to ibotenic acid. IboD, a putative decarboxylase, was proposed to catalyse the final conversion of ibotenic acid to muscimol [18]. These downstream assignments were inferred rather than individually demonstrated by purified-enzyme assays, and alternative reaction sequences remain possible.

Recent phylogenomic work considerably expands the taxonomic context of this pathway. Su et al. screened 25 Amanita genomes using the seven *A. muscaria ibo* genes as queries and identified 134 *ibo* homologues distributed among 21 species of section Amanita [43]. All seven genes were recovered from 17 species; four additional species yielded incomplete sets, which the authors considered likely to reflect limitations of the available genomic material rather than necessarily genuine gene loss. The *ibo* BGC occurred within a major monophyletic clade, and molecular dating placed its origin at approximately 28 million years ago [43].

Genomic presence, however, is not equivalent to demonstrated metabolite production. Among the species carrying *ibo* genes, only a subset has also been confirmed analytically to contain IA and MUS, and non-detection of either genes or metabolites can be affected by genome completeness, specimen selection, developmental stage, analytical sensitivity, and other methodological factors [8,43]. The phylogenomic findings therefore strengthen evidence for the evolutionary distribution of biosynthetic capacity but do not establish quantitative pathway activity in every species. This distinction is considered further in Section 3.4.

**Figure 2:**
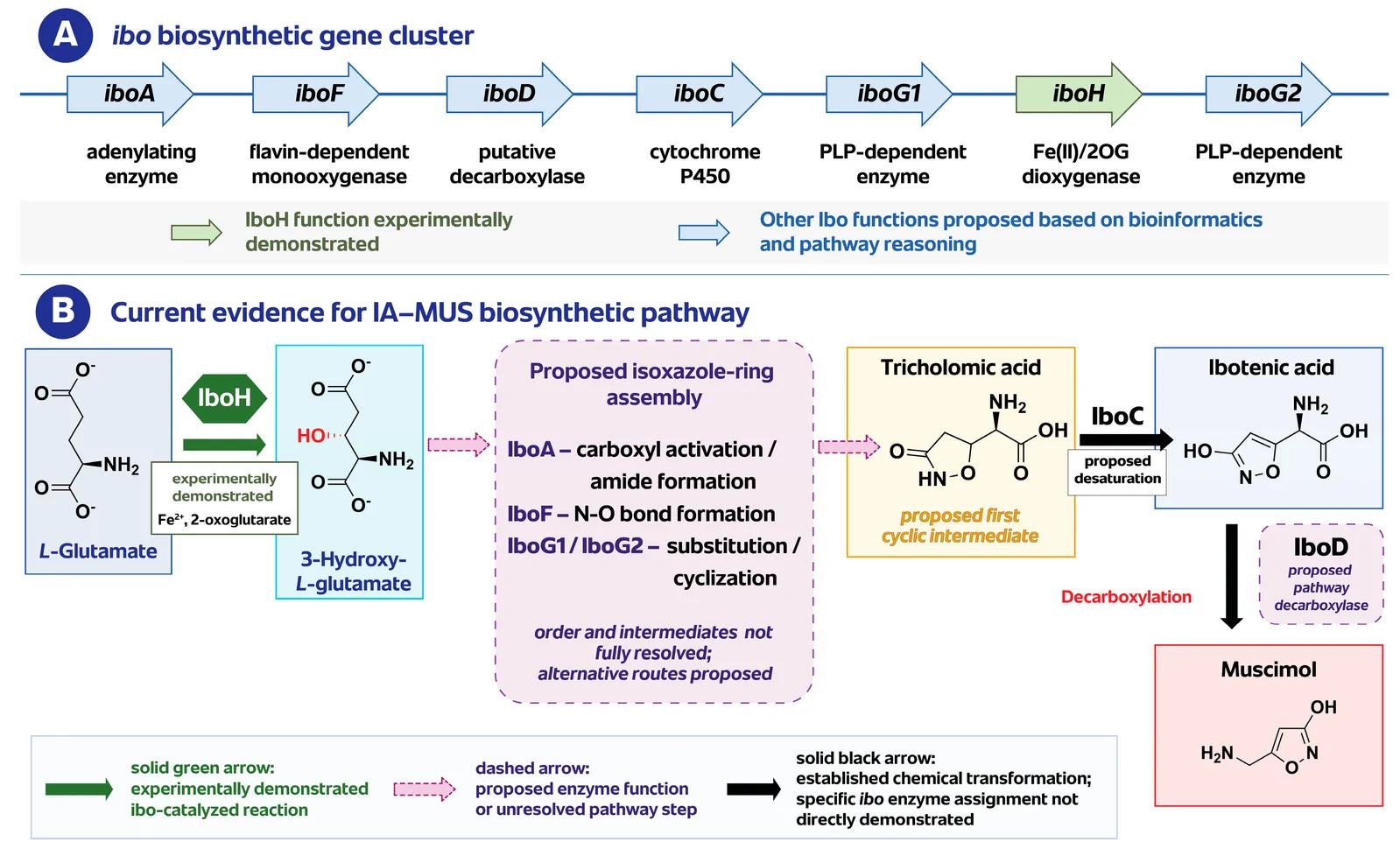
The *ibo* biosynthetic gene cluster and current evidence for ibotenic acid and muscimol biosynthesis. The *A. muscaria ibo* cluster comprises *iboA*, *iboF*, *iboD*, *iboC*, *iboG1*, *iboH*, and *iboG2*. Hydroxylation of *L*-glutamate to 3-hydroxy-*L*-glutamate by IboH is the experimentally demonstrated first committed step [18]. The proposed functions of IboA, IboF, IboG1/IboG2, IboC, and IboD are based primarily on sequence similarity, genomic and transcriptomic association, and biosynthetic reasoning; the order of the intermediate reactions remains incompletely resolved. Tricholomic acid has been proposed as the first cyclic intermediate, followed by IboC-mediated desaturation to ibotenic acid. Decarboxylation of ibotenic acid to muscimol is chemically established, whereas assignment of this step specifically to IboD remains a proposed pathway function. Recent phylogenomic analysis detected *ibo* genes in 21 species of *Amanita* section *Amanita*, supporting a broader evolutionary distribution of the biosynthetic capacity without establishing metabolite abundance in every species [43].

The pathway also provides a molecular framework for studies of biological regulation, but it should not be used as evidence that nutrient availability or environmental stress controls IA production. Demonstrating regulation would require measurements of pathway expression, enzyme activity, metabolic flux, or metabolite output under controlled conditions. The current evidence for individual pathway steps and the distinction between experimentally demonstrated and proposed enzyme functions are summarized in Figure 2.

### 3.3. Formation of Muscimol

Muscimol is formed by decarboxylation of ibotenic acid. Within the proposed *ibo* pathway, IboD is the leading candidate for the corresponding decarboxylase, although this specific enzymatic assignment has not yet been established by direct biochemical characterization [18]. Its concentration in a sample may therefore reflect both biological formation and chemical transformation after collection. This dual origin complicates interpretation.

A high muscimol concentration does not necessarily indicate increased fungal biosynthesis. It may instead result from partial conversion of ibotenic acid during drying, heating, extraction, or storage. Conversely, a low ibotenic acid concentration may reflect decarboxylation, degradation, leaching, incomplete extraction, or genuine biological variation. Studies reporting only one of the two compounds provide an incomplete description of the chemical profile.

The IA/MUS ratio is used throughout this review as a descriptive measure of relative analyte abundance and is defined as

**RIA/MUS = CIA/CMUS**

where CIA and CMUS are the concentrations of ibotenic acid and muscimol measured in the same sample, expressed in identical units and normalized to the same mass- or volume-based denominator. Under these conditions, the ratio is dimensionless. For studies evaluating IA-to-MUS transformation or mass balance, however, IA and MUS should be expressed as molar quantities because the two compounds have different molecular masses. Ratios in which the MUS concentration lies close to the LOQ should be regarded as analytically unstable and interpreted together with the corresponding measurement uncertainty. A numerical ratio is meaningful only when both analytes are quantified reliably. If either concentration is below the applicable LOQ, a value of zero should not be substituted for calculation; the ratio should instead be reported as not quantifiable or, where analytically justified, as a bound based on the relevant detection or quantification limit.

The IA/MUS ratio should always be accompanied by the underlying absolute concentrations because similar ratios can occur in samples with markedly different total analyte burdens. It is therefore a secondary descriptor of sample composition rather than an independent measure of toxicity.

### 3.4. Taxonomic Distribution: Metabolite Detection Versus Biosynthetic Capacity

Ibotenic acid and muscimol are not distributed uniformly across the genus *Amanita*, but taxonomic distribution needs to be considered at two distinct evidential levels: direct chemical detection of the metabolites and genomic evidence for the capacity to synthesize them.

The most extensive targeted chemical survey remains that of Su et al., who analysed 84 specimens representing 24 species of *Amanita* section *Amanita* using UPLC–MS/MS, as designated in the original study [8]. IA and MUS were detected in 10 species, with reported concentrations ranging from approximately 0.61 to 32.1 g/kg dry weight for IA and from 0.0056 to 5.87 g/kg dry weight for MUS [8]. The upper IA value corresponds to approximately 3.2% of dry mass. This study provides direct evidence of interspecific differences within the section, but it remains a single large regional survey and does not establish a fixed chemical phenotype for every species or population.

More recent taxonomic work has extended the analytical dataset. Su et al. described two new species from China and screened dried specimens by LC–MS/MS [44]. IA and MUS were detected in *A. parvisychnopyramis*, at 0.71–4.04 g/kg and 2.55–2.68 g/kg, respectively, whereas neither analyte produced a corresponding signal in the analysed *A. flavomelleiceps* material [44]. These observations add direct species-level chemical evidence, but the negative result should remain condition-specific. The specimens had undergone defined drying and subsequent storage before analysis, and analytical non-detection cannot by itself establish that a species is biologically incapable of producing the compounds.

A different picture emerges from genomic evidence. The 2026 phylogenomic analysis by Su et al. searched 25 *Amanita* genomes using the seven known *ibo* genes and identified 134 homologues [43]. ibo genes were detected in 21 species; all seven genes were recovered from 17 species, whereas only subsets were obtained from four additional species, most likely because of limitations in the available DNA or genome assemblies [43]. The ibo-positive species formed a major monophyletic lineage within section Amanita, supporting a broader evolutionary distribution of biosynthetic capacity than can presently be demonstrated by targeted toxin measurements alone.

Presence of the *ibo* cluster should not, however, be equated with measurable IA or MUS production. A genomic sequence establishes biosynthetic potential, not pathway expression, enzyme activity, metabolic flux, or metabolite abundance at the time of sampling. Conversely, analytical non-detection may reflect developmental stage, anatomical compartment, sample amount, hydration, post-harvest treatment, extraction recovery, analytical sensitivity, or concentrations below the LOD or LOQ. Incomplete genome assemblies may introduce the opposite problem by creating an apparent absence of individual *ibo* genes. Thus, disagreement between genomic and analytical surveys is not necessarily biological contradiction.

Intraspecific genomic and metabolomic evidence adds another layer. Nickles et al. found broad conservation of biosynthetic gene clusters and untargeted molecular families among European and introduced South African *A. muscaria* populations [45]. Their study sequenced 24 Northern- and Southern-Hemisphere specimens and found only 13 of 273 molecular families to be unique to South African material [45]. This supports broad conservation of specialized-metabolite capacity across the sampled populations, but it was not a targeted quantitative survey of IA and MUS and should not be interpreted as demonstrating geographic equivalence of their concentrations.

Taxonomic interpretation depends on the type of evidence available. Targeted detection of IA and MUS provides direct evidence that the compounds were present in the analysed material under the stated conditions. Detection of the *ibo* BGC provides direct genomic evidence of biosynthetic capacity but not quantitative metabolite production. Clinical poisoning patterns or phylogenetic proximity may support prioritization of species for investigation, but neither substitutes for chemical confirmation. On the current evidence, both metabolite occurrence and biosynthetic capacity are concentrated within *Amanita* section *Amanita*, while the precise correspondence between genotype and chemical phenotype remains incompletely mapped.

The chemical relationships, established pharmacological properties, and main interpretative limitations of the principal compounds are summarized in Table 1.

Taken together, the chemical relationship between ibotenic acid and muscimol and the glutamate-derived entry point of ibotenic acid biosynthesis are well established. Direct evidence supports interspecific variation within *Amanita* section *Amanita*, but the taxonomic dataset remains limited in terms of independent studies. Processing-related transformation is supported separately by experiments conducted under defined conditions. By contrast, environmental regulation of the pathway, predictable clinical interpretation of the IA/MUS ratio, and uniform conversion during domestic or commercial processing remain unconfirmed.

## 4. Evidence for Biological Sources of Variability

### 4.1. Taxonomic Variation

The clearest direct evidence for taxonomic variation comes from the comparative study by Su et al., who analysed 84 specimens representing 24 species of *Amanita* section *Amanita* by UPLC–MS/MS [8]. IA and MUS were detected in 10 species. This is an important dataset because the species were examined within a common analytical framework, but it should not be mistaken for a large body of independent evidence. At present, conclusions about taxonomic distribution rely heavily on this single comparative study.

The results demonstrate interspecific differences within section Amanita, not a uniform concentration pattern for *A. muscaria*. Detection in one species cannot be used to predict concentrations in another, and even within a species the measured values may vary with maturity, tissue selection, geographic origin, sample handling, and analytical recovery.

Species identification is therefore a fundamental requirement in comparative studies. Morphological identification alone may be insufficient for immature, damaged, processed, or closely related specimens. Molecular confirmation should be considered when taxonomic uncertainty could affect chemical or toxicological interpretation [46].

Reports describing material only as “fly agaric”, “*Amanita* mushroom”, or a commercial *Amanita* preparation should not be treated as equivalent to chemically characterized and taxonomically authenticated *A. muscaria* specimens.

### 4.2. Developmental Stage

Developmental variation has been examined directly, but only in a small number of studies. An early study by Tsunoda et al. followed *A. muscaria* fruiting bodies through maturation and analysed IA and MUS in the cap, stipe, base, and whole fruiting body [9]. Mean concentrations for the whole fruiting body were 343 ppm IA and 22 ppm MUS; these values refer to the mass of the non-dried fruiting-body material used for analysis rather than to a dry-weight-normalized concentration [9]. The cap contained the highest mean concentrations of both analytes, followed by the base and stipe. During maturation, the direction of change differed among anatomical parts, whereas concentrations calculated for the whole fruiting body remained comparatively stable [9].

A more recent study by Su et al. found lower IA and MUS concentrations in mature than in less developed specimens of *A. subglobosa* [8]. This result provides independent evidence that maturation can influence the isoxazole profile within *Amanita* section Amanita, but it does not reproduce the earlier *A. muscaria* pattern and should not be extrapolated to that species without qualification.

Taken together, these two datasets show why developmental stage and anatomical sampling need to be recorded, but they do not establish a single maturation trajectory for IA or MUS. Concentration changes may reflect altered biosynthesis, redistribution among tissues, tissue expansion, hydration, degradation, or conversion of IA to MUS. In particular, a decline expressed per gram of fresh tissue cannot by itself be interpreted as reduced biosynthetic activity.

Future comparisons would benefit from replicated sampling at defined developmental stages, with separate reporting of anatomical compartments, whole-fruiting-body values, moisture content, and both fresh- and dry-weight concentrations.

### 4.3. Tissue-Specific Variation

Anatomical parts of a fruiting body are not necessarily chemically equivalent. Caps, stipes, gills, surface tissues, and internal tissues differ in physiological function, water content, developmental state, and exposure to microorganisms and physical damage.

A second line of evidence comes from the metabolomic study of Deja et al., who found broader low-molecular-weight differences between caps and stipes of *A. muscaria* [47]. The study also examined associations between metabolomic composition and topsoil type. These findings support anatomically standardized sampling, but they should not be interpreted as direct proof that ibotenic acid or muscimol consistently accumulates in a particular tissue. The study evaluated broader metabolomic variation rather than establishing a reproducible tissue-distribution pattern for the two isoxazole compounds.

Studies that analyze whole fruiting bodies cannot be directly compared with those examining caps, stipes, or selected tissue fractions. Homogenizing entire specimens may also conceal local differences. Meaningful comparison requires clear identification of the anatomical material and whether the analysed sample represents a single fruiting body, pooled material, or a subsample of homogenized tissue.

Direct mapping of ibotenic acid and muscimol across defined anatomical compartments remains limited. Such work would require multiple fruiting bodies collected at comparable developmental stages, separate analysis of the cap, gills, stipe, and outer tissues, and correction for differences in moisture content.

### 4.4. Variation Among Individual Specimens and Populations

Substantial variation may occur among individual fruiting bodies, including specimens assigned to the same species and collected from the same general region. Potential biological contributors include genotype, developmental history, tissue composition, physiological condition, and the time elapsed between collection and analysis.

The study of Tsunoda et al. provides direct evidence of specimen-level variability in *A. muscaria*. Among 62 fruiting bodies, IA and MUS concentrations varied substantially, while the authors found no consistent effect of lone versus colonial growth or fruit-body size on either analyte [9].

Geographic differences reported across studies should be interpreted with particular caution. Samples collected in different countries are frequently analyzed using different extraction procedures, analytical platforms, calibration approaches, and reporting units. Documented inter- and intracontinental phylogeographic structure within the *A. muscaria* complex provides an additional reason to avoid treating geographically separated material as chemically uniform [48].

A recent genomic and untargeted metabolomic comparison of European and introduced South African *A. muscaria* populations found broad conservation of biosynthetic gene clusters and molecular families; only 13 of 273 molecular families were unique to the South African material [45]. Because the study did not establish IA- and MUS-specific concentration patterns, it supports caution rather than a direct conclusion about geographic variation in these two analytes.

Evidence for population-level variation would be stronger if studies used replicated sampling from several locations, molecular species confirmation, standardized developmental staging, identical tissue compartments, and a common validated analytical method. Such designs remain uncommon. The current literature therefore supports specimen-to-specimen variability but does not yet permit robust geographic mapping of IA and MUS concentrations in *A. muscaria*.

### 4.5. Hydration and Concentration Units

Water content is a major source of uncertainty in comparisons of fungal metabolites. Fresh fruiting bodies contain substantial and variable amounts of water, which change during maturation, environmental exposure, transport, and storage. Concentrations expressed per gram of fresh weight may therefore differ even when the absolute amount of analyte per unit of dry matter is similar.

Drying removes water but can also alter the analytes through decarboxylation or degradation. Dry-weight normalization does not by itself solve this problem when the drying procedure has already changed the chemical composition. The drying temperature, duration, airflow, light exposure, and residual moisture must therefore be reported alongside the concentration units.

Concentration data are interpretable only when their normalization basis is clear. Tissue, developmental stage, and moisture or dry-matter content determine how apparently similar numerical values can be compared, while absolute IA and MUS concentrations remain necessary for interpretation of the chemical profile.

The IA/MUS ratio is meaningful only when interpreted alongside the underlying concentrations; similar ratios can describe samples with very different total analyte burdens.

The biological evidence is informative but rests on relatively few independent datasets. Interspecific differences are demonstrated most clearly by one large comparative survey of *Amanita* section *Amanita* [8]. Developmental evidence consists mainly of the earlier *A. muscaria* study [9] and the *A. subglobosa* observations reported by Su et al. [8]. Tissue-level heterogeneity is supported by the same older *A. muscaria* dataset and by broader metabolomic work [47], but modern replicated mapping of IA and MUS across anatomical compartments is still lacking. Geographic patterns are even less certain because differences between studies are easily confounded by sampling and analytical methodology. Table 2 summarizes the primary datasets underlying these conclusions and reports the species, sample size, analytical approach, and claim-level evidence category for each source of biological variability.

## 5. Post-Harvest Transformation and Stability

### 5.1. General Considerations

The chemical composition of *Amanita muscaria* may change substantially after collection. Drying, heating, cooking, extraction, storage, and exposure to light can affect the absolute concentrations of ibotenic acid and muscimol as well as their relative proportions. These changes are relevant to analytical interpretation because the measured profile may no longer represent the composition of the fresh fruiting body.

**Figure 3:**
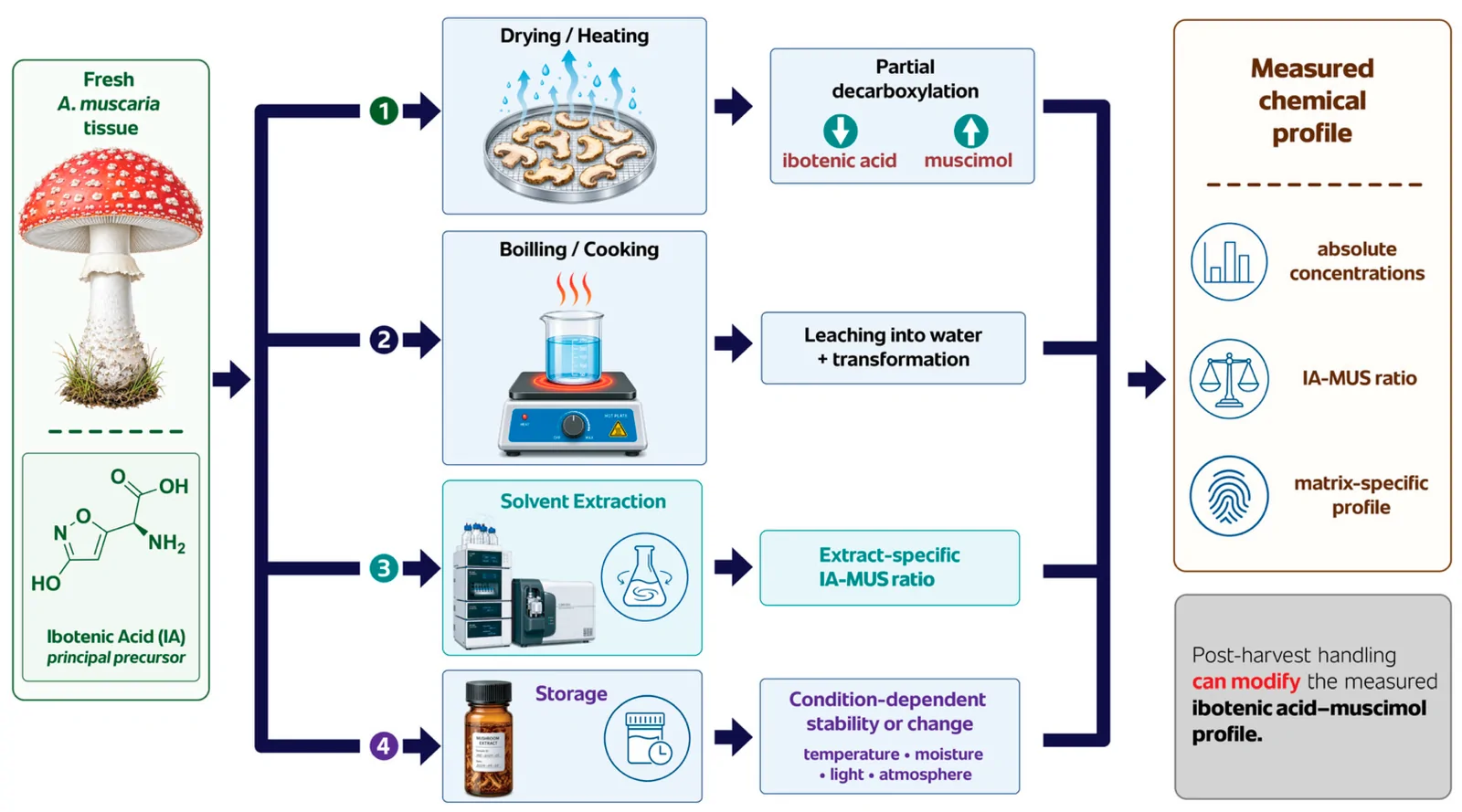
Principal post-harvest factors affecting the measured ibotenic acid–muscimol profile. Drying and heating may promote decarboxylation and water loss, cooking may combine chemical transformation with leaching, and extraction may produce solvent- and pH-dependent recovery. Storage effects are condition-dependent: direct evidence supports stability under specific dry/cool/dark and salted/refrigerated conditions, whereas other storage regimes remain incompletely characterized [10]. Interpretation therefore requires absolute IA and MUS concentrations together with complete sample-handling information. Evidence concerning the principal processing routes is summarized from [10,11,15].

The principal post-harvest routes that may alter the measured ibotenic acid–muscimol profile are summarized in Figure 3.

Post-harvest and biological variation are analytically distinct, even though they may produce similar differences between samples. A difference between two samples may arise from species, developmental stage, or tissue composition, but it may also be introduced after collection through sample handling. Studies that do not describe processing and storage conditions cannot reliably attribute observed differences to biological or ecological factors.

The term “conversion” also requires caution. A decrease in ibotenic acid accompanied by an increase in muscimol is consistent with decarboxylation, but incomplete mass balance may indicate additional processes, including leaching, degradation, variable extraction recovery, or formation of minor products. Measurements of both compounds are therefore necessary, preferably together with total analyte recovery.

### 5.2. Drying and Thermal Treatment

Drying is one of the most frequently discussed post-harvest factors affecting the ibotenic acid–muscimol profile. Heat can promote decarboxylation of ibotenic acid to muscimol, while removal of water changes the concentration basis on which results are reported. These processes may occur simultaneously and should not be conflated.

Tsunoda et al. reported that sun-drying and heater-drying of *A. muscaria* reduced ibotenic acid and increased muscimol, supporting processing-dependent conversion [10]. This finding provides direct evidence that drying can alter the chemical profile. However, it does not justify the general claim that drying produces complete or reproducible conversion. The outcome depends on temperature, duration, airflow, sample thickness, initial water content, pH, and the physical condition of the tissue.

Comparison between fresh and dried material is further complicated by water loss. A concentration expressed per gram of dried material may appear higher even when the absolute amount of analyte has decreased.

Interpretation of drying experiments depends on the mass balance as well as the measured concentrations. Initial and final sample mass, thermal conditions, residual moisture, and the normalization basis therefore need to accompany IA and MUS results. The complete reporting framework is summarized later in Section 10.1.

Thermal treatment should not be treated as a single standardized process. Low-temperature dehydration, oven drying, sun exposure, roasting, and prolonged heating differ in their capacity to promote decarboxylation or degradation. Results from one procedure cannot be generalized to another without direct analytical confirmation.

### 5.3. Cooking and Leaching

Cooking may alter the chemical profile through several mechanisms. Heat can promote decarboxylation, whereas contact with water may remove polar compounds from fungal tissue by leaching. The relative importance of these processes depends on the preparation method [10].

Boiling, simmering, roasting, and preparation in acidic or alkaline media are chemically distinct. During boiling, ibotenic acid and muscimol may migrate into the cooking water. A reduction in the concentration measured in the mushroom tissue does not necessarily indicate chemical degradation if the analytes are recovered in the liquid phase. Conversely, analysis of the cooking water alone may overestimate consumer exposure when part of the liquid is discarded.

Controlled cooking experiments are most informative when both the treated tissue and the cooking liquid are analysed. Temperature, treatment time, pH, water-to-sample ratio, tissue size, and pre-treatment concentrations are needed to reconstruct the fate of the analytes. Without measurements of both fractions, loss by leaching cannot be separated reliably from chemical conversion or degradation.

Without a mass-balance approach, it is difficult to distinguish conversion from leaching or analytical loss. Claims that a particular domestic preparation method “detoxifies” or “activates” *A. muscaria* should therefore be avoided unless supported by validated quantitative data.

### 5.4. Extraction-Dependent Chemical Profiles

Extraction does not simply transfer all fungal constituents into solution in their original proportions. Recovery of ibotenic acid and muscimol depends on solvent composition, pH, temperature, extraction time, sample-to-solvent ratio, and the number of extraction cycles.

Hydroalcoholic, aqueous, acidic, and other extraction systems may generate substantially different chemical profiles. Dushkov et al. quantified ibotenic acid, muscimol, and ergosterol in a hydroalcoholic extract of *A. muscaria*, illustrating that an extract should be regarded as a distinct preparation rather than as a direct equivalent of whole mushroom material [15].

Extraction conditions can also change the IA/MUS ratio independently of the original fungal composition. Differential recovery, pH-dependent transformation, prolonged processing, or heat generated during extraction may influence the final result. For this reason, studies of extracts should report extraction recovery for both analytes and should not assume that the measured ratio reflects that of the starting tissue.

This issue is particularly important for commercial products. Concentrated extracts, gummies, powders, and liquid formulations may undergo multiple extraction and formulation steps. Their composition cannot be inferred from the species name or from analytical data obtained for fresh fruiting bodies.

### 5.5. Storage, Moisture and Light Exposure

Storage has been examined directly under defined conditions. Tsunoda et al. reported that IA and MUS remained comparatively stable in dried *A. muscaria* stored in a cool, dark place for up to 90 days [10]. In a separate experiment, mushroom material was boiled, adjusted to 10% NaCl, and stored at 5 °C; both analytes remained stable over the observation period extending to 136 days [10]. These findings provide direct evidence of stability under the specific dry/cool/dark and salted/refrigerated conditions tested, but they should not be generalized to all stored mushroom preparations.

Stability under other conditions is less well characterized. Residual moisture, elevated temperature, oxygen exposure, light, packaging atmosphere, and the physical form of the sample may influence either chemical stability or analytical recovery, but their effects have not been quantified systematically enough to support a general claim that stored or dried *A. muscaria* is intrinsically unstable. Storage conditions should therefore be reported explicitly when samples from different studies are compared.

Light exposure requires separate consideration from storage duration itself. The light-associated formation of muscazone indicates that ibotenic acid can undergo photochemical transformation in addition to decarboxylation [1,41,42]. The detailed mechanism remains incompletely resolved, and muscazone formation should not be interpreted as evidence that all stored material undergoes progressive degradation. Storage time, temperature, light exposure, residual moisture, packaging conditions, and sample form are consequently relevant descriptors of the analysed material.

### 5.6. Freeze–Thaw and Sample Homogenization

The stability of ibotenic acid and muscimol during repeated freeze–thaw cycles has not been adequately characterized. Freeze–thaw history, homogenization method, particle size, and the time between thawing and extraction should therefore be reported as potential sources of analytical variability.

### 5.7. Analytical Implications and Evidence Limitations

Post-harvest handling can alter both sample composition and the analytical profile ultimately reported. A muscimol-rich result, for example, may reflect the original material, partial decarboxylation during drying or heating, differential extraction, leaching, or subsequent storage. The IA–MUS profile alone cannot distinguish among these possibilities.

Direct evidence supports procedure-specific changes during drying, heating, cooking and leaching, as well as stability under the two defined storage regimes examined by Tsunoda et al. [10]. The principal uncertainty concerns whether these findings extend to other storage environments, including variable humidity, elevated temperature, repeated light or oxygen exposure, different packaging systems, powdered preparations, and freeze–thaw cycles. Neither universal stability nor universal instability of stored *A. muscaria* should therefore be assumed without condition-specific data [10,15,40].

## 6. Analytical Determination: Study-Level Comparison of Methods

### 6.1. Analytical Challenges

Quantitative determination of ibotenic acid and muscimol is complicated by their low molecular mass and pronounced pH-dependent ionization. The ionic and zwitterionic forms discussed in Section 3.1 help explain their strong affinity for aqueous media, weak retention in many conventional reversed-phase systems, and sensitivity to mobile-phase composition and pH [33,36,37].

A second difficulty is analyte instability. Ibotenic acid may undergo decarboxylation or degradation during drying, storage, extraction, and instrumental preparation. The measured IA–MUS profile may therefore reflect both the original sample composition and changes introduced before analysis. Stability during extraction, autosampler storage, freeze–thaw cycles, and long-term storage must be assessed when the IA/MUS ratio is used as an analytical endpoint.

Matrix composition varies markedly among fresh fruiting bodies, dried powders, aqueous or hydroalcoholic extracts, serum, urine, gastric contents, and commercial products. A method validated for one matrix cannot be assumed to provide equivalent recovery, selectivity, or ionization efficiency in another. Method suitability must therefore be evaluated in relation to the intended application rather than inferred from the analytical platform alone.

Accordingly, the comparison below is organized around analytical purpose and matrix: quantitative profiling of fungal material, exposure confirmation in biological fluids, rapid screening, and analysis of heterogeneous commercial products. Instrumental platform is considered together with sample preparation, calibration, internal-standard strategy, sensitivity, validation characteristics, and intended evidential use. Numerical study-level performance is compared below on a study-by-study basis.

### 6.2. LC–MS/MS and HILIC–LC–MS/MS

Liquid chromatography coupled with tandem mass spectrometry provides selective identification and quantitative determination through the combined use of chromatographic retention and compound-specific ion transitions. It has been applied to mushroom tissue, extracts, serum, and multi-analyte toxicological screening [12,16,17,49].

Earlier ion-interaction HPLC methods also enabled simultaneous determination of ibotenic acid and muscimol, although their retention, detection, and sample-preparation requirements differ from those of current tandem-MS workflows [50].

Among the direct LC–MS/MS methods for fungal material, the study by Gonmori et al. provides one of the more complete quantitative datasets [12]. Mushroom material was homogenized in water/methanol (1:1), followed by anion-exchange solid-phase extraction on an Oasis MAX cartridge. Acivicin was used as the internal standard, and IA and MUS were separated on a TSK-GEL Amide-80 HILIC column without derivatization. Quantification by MRM was linear from 10 to 500 μg/g for both analytes (r ≥ 0.99), with reported recoveries of 84.6–107% [12]. The chromatographic program extended to 10 min. This combination of direct analysis, an internal standard, and matrix-specific recovery data makes the method particularly useful as a reference for quantitative work on mushroom material, although its performance cannot be transferred automatically to biological fluids or formulated products.

The main analytical advantage of tandem MS in this setting is not the instrument label itself but the ability to combine chromatographic retention with compound-specific ion transitions. For IA and MUS, this becomes particularly important when matrix components interfere with weakly retained polar analytes. Conversely, poor extraction recovery or inadequate chromatographic control cannot be corrected simply by using tandem-MS detection.

LC–MS/MS is therefore well suited to confirmatory analysis, forensic toxicology, controlled processing studies, and research requiring reliable quantification. Its performance, however, must be demonstrated through matrix-specific validation rather than assumed from the use of tandem mass spectrometry.

### 6.3. UHPLC–MS/MS in Comparative Fungal Studies

UHPLC–MS/MS is particularly useful when a common analytical workflow is applied to a large series of fungal samples. Su et al., using the designation UPLC–MS/MS in the original study, analysed 84 specimens representing 24 species of *Amanita* section *Amanita* [8]. Among toxin-positive species, IA concentrations ranged from approximately 0.61 to 32.1 g/kg dry weight and MUS concentrations from 0.0056 to 5.87 g/kg [8]. The original paper reports several sample means to four decimal places, but this numerical resolution should not be interpreted as equivalent analytical precision. For example, the highest IA result, measured in *A. sychnopyramis*, was reported as 32.0932 ± 12.71947 g/kg [8].

The study is important primarily as a large comparative application rather than as evidence that UPLC is intrinsically superior to other LC formats. Analytical throughput, short chromatographic runs, and efficient separation are advantageous for replicated taxonomic or developmental studies, but evidential strength still depends on calibration, recovery, matrix effects, analyte stability, and consistent sample preparation. For this reason, the study-level comparison distinguishes method-validation studies from studies in which an established platform was applied principally to biological comparison.

### 6.4. Capillary Electrophoresis and CE–ESI-MS/MS

Capillary zone electrophoresis provides a chromatographically independent approach to IA and MUS determination. Poliwoda et al. developed a CZE method for *A. muscaria*, *A. pantherina*, and *A. citrina* in which the analytes were extracted by ultrasound using methanol and 1 mM sodium phosphate buffer at pH 3 (1:1, *v*/*v*) [13]. Separation was performed in a 25 mM phosphate buffer containing 5% acetonitrile. The calibration range extended from 2.5 to 7000 μg/mL for both analytes, recoveries exceeded 87%, and the reported intraday and interday variation was 1.0% and 2.5% RSD, respectively [13]. Identification relied on migration time, UV spectra, and standard addition.

The method demonstrates that tandem MS is not indispensable for quantitative work on relatively concentrated fungal extracts. Its trade-off is lower molecular specificity than MS-based confirmation, which becomes more important in complex biological or formulated matrices.

The main limitations of conventional CE are lower concentration sensitivity in some configurations, limited injection volume, and sensitivity of migration times to capillary condition, buffer composition, temperature, and electroosmotic flow. Reproducibility may be particularly challenging in complex mushroom or biological matrices unless capillary conditioning and sample cleanup are tightly controlled.

Coupling CE with tandem MS markedly improves molecular confirmation. Ginterová et al. developed a CE–ESI–MS/MS method for IA, MUS, and muscarine and tested it in spiked human urine [14]. The reported LODs were 0.15 ng/mL for IA, 0.05 ng/mL for MUS, and 0.73 ng/mL for muscarine. Migration-time RSDs ranged from 0.93% to 1.60%, while peak-area RSDs ranged from 2.96% to 3.42%; calibration coefficients exceeded 0.999 [14]. The method required little sample pretreatment, although it was evaluated in spiked urine rather than through a large series of clinical specimens.

CE–ESI-MS/MS remains more specialized than routine LC–MS/MS. Interface stability, migration-time reproducibility, and availability of instrumentation may restrict its broader implementation. It is best considered a complementary or application-specific technique rather than a universally interchangeable alternative.

### 6.5. Biological Matrices: Matrix-Specific Performance

Biological matrices require a different analytical scale from mushroom material. Concentrations are lower, endogenous components are more prominent, and the interval between exposure and sampling becomes part of the interpretation. This is reflected in the validation characteristics of the available methods.

Hasegawa et al. adapted the HILIC–LC–MS/MS workflow used for mushrooms to human serum [51]. A 100 μL serum aliquot was processed by Oasis MAX solid-phase extraction with acivicin as internal standard. The method was linear from 10 to 1000 ng/mL for both IA and MUS (r ≥ 0.999), with LODs of 1.0 ng/mL for IA and 2.5 ng/mL for MUS and recoveries of 87.9–103% [51]. The need to re-optimize extraction for serum is itself instructive: performance obtained with fungal material did not transfer directly to the biological matrix.

Xu et al. pursued a different strategy for plasma, using acetonitrile protein precipitation followed by bimolecular dansylation and LC–triple-quadrupole MS [17]. Isotopically labelled L-tyrosine-13C9,15N and tyramine-d4 were used as internal standards for IA and MUS, respectively. The linear ranges were 1–500 ng/mL for IA and 1–200 ng/mL for MUS, with LODs of 0.3 and 0.1 ng/mL. Recoveries were 90.7–111.4% for IA and 85.1–94.2% for MUS, with corresponding RSDs of 6.4–10.3% and 5.0–8.9% [17]. Derivatization increased analytical sensitivity but also introduced additional sample-preparation steps.

Urine has been analysed using still different approaches. Stříbrný et al. isolated IA and MUS on a strong cation exchanger, derivatized them with ethyl chloroformate, and quantified the derivatives by GC–MS using cycloserine as internal standard [49]. Their calibration series covered 1–15 μg/mL, and the reported LOD was 1 μg/mL for both compounds. Ginterová et al. subsequently demonstrated substantially lower detection limits with CE–ESI–MS/MS in spiked urine [14]. Deja et al. used ^1^H NMR spectroscopy with a 1D-NOESY pulse sequence for rapid determination of IA and MUS in human urine [52], illustrating that urine analysis is not restricted to chromatographic or electrophoretic MS techniques.

These studies show why biological-fluid methods should be compared within the same matrix rather than against mushroom assays. A lower numerical LOD does not by itself establish a superior method if extraction, confirmation criteria, sample volume, clinical timing, or validation scope differ.

### 6.6. Rapid and Ambient-Ionization Mass Spectrometry

Rapid screening represents a different analytical objective from confirmatory quantification. Luo et al. recently evaluated paper-spray and direct-mushroom-spray MS approaches for mushroom toxins [53]. Direct spraying from mushroom tissue allowed selected toxins, including IA, to be identified within approximately 2–3 min. For quantitative IA analysis, the authors developed an anion-exchange modified paper-spray tandem-MS method (AEPS–MS/MS).

For IA in mushroom extract, the AEPS–MS/MS calibration range was 5–40 μg/mL, with an LOD of 1.3 μg/mL and an LOQ of 4.3 μg/mL [53]. Reported accuracy ranged from −14.0% to +5.1%, and precision from 10.8% to 18.0%. The method was considerably faster than conventional chromatographic workflows, but its sensitivity was lower than that of the HPLC–MS/MS comparator used in the same study. Its principal value is therefore rapid screening and targeted high-throughput analysis rather than replacement of chromatographic confirmation in every setting.

### 6.7. Commercial Products and Multi-Analyte Screening

Commercial products require separate matrix-specific treatment because gummies, chocolates, capsules, tinctures, and blended extracts contain excipients and may also have uncertain fungal identity. Targeted determination of IA and MUS and broader screening for unexpected or undeclared constituents answer different analytical questions. Multi-analyte LC–MS/MS or accurate-mass screening can therefore be useful when adulteration or mislabelling is a concern [54,55].

Analytical results obtained for formulated products should not be used to infer the natural chemical phenotype of authenticated *A. muscaria*. Recovery, matrix effects, calibration, product homogeneity, and batch identity need to be established for the finished matrix. The broader public health and regulatory implications of commercial products are considered in Section 9.

### 6.8. Calibration, Internal Standards, and Validation

Validation requirements depend on the matrix and intended use. For quantitative analysis in biological fluids, ICH M10 provides the current harmonized framework for bioanalytical method validation and study-sample analysis, including selectivity and specificity, calibration, accuracy, precision, carryover, dilution integrity, matrix effects, recovery, and analyte stability [56]. Its formal scope concerns bioanalysis of biological samples in clinical and applicable nonclinical studies; it should not be presented as a regulatory validation standard for mushroom tissue or food-like commercial matrices. For these matrices, analogous performance characteristics should instead be established on a fit-for-purpose basis.

Internal-standard selection is method-specific. Stable-isotope-labelled analogues can be advantageous when available because they may compensate for extraction and ionization variability, but published IA/MUS methods also use non-isotopic internal standards. The choice should therefore be justified and its limitations reported rather than treated as a universal requirement. Matrix-matched calibration is particularly important when extraction recovery or ionization differs among mushroom tissue, biological fluids, extracts, and formulated products [57,58].

Stability experiments should reproduce the conditions relevant to the actual study samples. For IA, this includes the possibility of transformation during preparation or storage, so stability assessment needs to distinguish analytical change from biological variation. Missing matrix-effect or stability data should be reported as a validation limitation rather than interpreted as evidence that the method itself is intrinsically unreliable.

### 6.9. Cross-Study Comparability

Numerical performance can be compared meaningfully only within an appropriate matrix and analytical purpose. An LOD expressed in ng/mL for plasma cannot be ranked directly against one reported in µg/g of mushroom material, while recovery and precision depend on extraction, fortification level, calibration model, and matrix composition. Analysis time also has to be interpreted in context: a rapid screening procedure and a confirmatory quantitative method are designed to answer different questions.

For this reason, the published analytical characteristics are compared study by study rather than converted into a single platform ranking. NR is used when a parameter was not reported in a directly comparable form. The IA/MUS ratio remains complementary to absolute quantification and does not correct for differential recovery, matrix effects, analyte conversion, or differences in sample normalization.

### 6.10. Study-Level Comparison of Analytical Methods

The published methods differ not only in analytical platform but also in matrix, sample preparation, derivatization, calibration range, and validation depth. Table 3 therefore compares individual studies rather than broad technique categories. Numerical performance values are reproduced only where they were reported by the original authors and should be read in the context of the matrix and analytical purpose for which each method was developed.

## 7. Pharmacological and Toxicological Significance

### 7.1. Pharmacological Basis

Ibotenic acid and muscimol differ substantially in their principal pharmacological actions. Ibotenic acid is structurally related to glutamate and acts as an agonist at NMDA receptors and at group I and II metabotropic glutamate receptors [6,38,39]. Experimental evidence indicates that its excitotoxic lesioning effect is principally associated with NMDA-receptor activation, whereas activation of metabotropic glutamate receptors can be dissociated from this neurotoxicity [6,38]. Muscimol, in contrast, is a potent agonist of ionotropic GABA receptors and reduces neuronal excitability through direct activation of inhibitory neurotransmission [7].

These established receptor-level differences provide a mechanistic basis for the mixed neurological manifestations associated with *Amanita muscaria* intoxication. They do not, however, permit individual symptoms to be assigned exclusively to one compound. Human exposure usually involves both metabolites, uncertain doses, a complex fungal or product matrix, and possible chemical transformation before ingestion.

Experimental pharmacology and clinical toxicology should therefore be distinguished. Ibotenic acid-induced neuronal lesions in laboratory models demonstrate excitotoxic potential under controlled exposure conditions, but they are not equivalent to the usual pattern of acute mushroom poisoning. Similarly, the inhibitory activity of purified muscimol does not mean that muscimol-containing preparations invariably produce a purely sedative syndrome.

### 7.2. Evidence from Experimental Studies

Experimental studies provide strong evidence for the receptor activity of purified ibotenic acid and muscimol. Ibotenic acid has been used to activate glutamatergic pathways and generate excitotoxic lesions in defined brain regions, whereas muscimol has been widely used to suppress neuronal activity through GABA-receptor activation [1,7,59].

A recent exploratory mouse study reported time- and region-dependent changes in several neurotransmitter-related measures after acute ibotenic acid exposure [59]. These findings indicate broader neurochemical perturbation than can be inferred from glutamatergic activity alone, but they do not establish a defined multi-receptor mechanism and should not be extrapolated directly to the clinical effects of mushroom ingestion.

### 7.3. Clinical Manifestations

Published case reports and poison-center data describe a heterogeneous syndrome following ingestion of *A. muscaria* and related isoxazole-containing mushrooms. Reported manifestations include nausea, vomiting, dizziness, confusion, agitation, hallucinations, delirium, impaired coordination, ataxia, somnolence, and altered consciousness [2,3,5,60]. Severe central nervous system depression, seizures, respiratory complications, and fatal outcomes have been reported, but appear considerably less common than transient neurobehavioral disturbance.

The occurrence of both excitatory and inhibitory features is consistent with exposure to ibotenic acid and muscimol. However, the clinical literature has important limitations. Many reports lack:

- Analytical confirmation of the ingested material;
- Quantitative measurement of ibotenic acid and muscimol;
- Reliable dose estimation;
- Complete information on preparation and storage;
- Exclusion of other mushroom species or substances;
- Standardized assessment of symptom severity.

Consequently, variability among clinical cases cannot currently be attributed specifically to differences in the IA/MUS ratio. Age, body mass, comorbidities, medications, co-ingestions, gastrointestinal absorption, and the time elapsed before medical assessment may be equally important.

### 7.4. Toxicological Interpretation of the IA–MUS Profile

The relative abundance of ibotenic acid and muscimol is pharmacologically relevant because the compounds act through different neurotransmitter systems. An ibotenic-acid-rich sample may be expected to provide a greater excitatory contribution, whereas a muscimol-rich sample may provide a greater inhibitory contribution. This interpretation is mechanistically reasonable but remains insufficiently validated in human intoxication.

No robust clinical dataset currently links analytically measured IA/MUS ratios with standardized symptom profiles, severity scores, or outcomes. Most clinical reports do not quantify both compounds in the consumed material or biological samples. The commonly proposed distinction between an “ibotenic-acid-dominant” excitatory syndrome and a “muscimol-dominant” inhibitory syndrome should therefore be presented as a hypothesis rather than a clinically established classification.

Several additional factors restrict ratio-based interpretation:

1. The ratio does not describe the total dose. Samples with the same ratio may contain markedly different absolute concentrations.
2. The analyzed material may not represent the ingested material. Remaining mushrooms, product fragments, serum, and urine reflect different stages of exposure and metabolism.
3. Processing may change both compounds before ingestion. Drying, heating, leaching, extraction, and storage can modify absolute concentrations and relative proportions.
4. Symptoms may change over time. Absorption and elimination of the two compounds may not occur at identical rates.
5. Co-exposures may dominate the clinical presentation. Alcohol, sedatives, stimulants, pharmaceuticals, or undeclared product ingredients can substantially modify toxicity.

The IA/MUS ratio should therefore be treated as one component of chemical characterization, not as an independent predictor of clinical outcome.

### 7.5. Quantitative Dose Context and Toxicokinetic Uncertainty

Published concentration data provide a useful order-of-magnitude reference, although they do not define a fixed dose per fruiting body. In the developmental study of *A. muscaria* by Tsunoda et al., the mean concentrations measured across whole fruiting bodies were 343 ppm for IA and 22 ppm for MUS [9]. These concentrations were reported relative to the mass of the non-dried fruiting-body material analysed in the original study, rather than on a dry-weight-normalized basis. On this same mass basis, the values correspond to approximately 0.343 mg IA and 0.022 mg MUS per gram of mushroom. Thus, 100 g of material on the same wet-weight basis would contain approximately 34.3 mg IA and 2.2 mg MUS at the reported mean concentrations. On a molar basis, 34.3 mg IA corresponds to approximately 0.217 mmol and would yield about 24.8 mg MUS if decarboxylation were complete. This value is a stoichiometric upper bound for conversion of the IA fraction rather than an estimate of the muscimol dose actually delivered by 100 g of mushroom, because conversion may be incomplete and processing may also cause leaching or degradation.

Human pharmacological data for purified muscimol are older but relevant to dose interpretation. In a double-blind, placebo-controlled study involving six patients with chronic schizophrenia, Tamminga et al. found that doses below 5 mg could produce a tranquilizing effect in some participants, whereas the higher-dose phase was associated with somnolence or diffuse myoclonic twitching and, in several subjects, vivid dreams, dizziness, or confusion [61]. Shoulson et al. administered oral muscimol to ten patients with Huntington’s disease in a double-blind study; the treatment did not improve motor or cognitive function overall, although neurological and behavioural changes were observed in some participants [62]. In a separate neuroendocrine study involving patients with Huntington’s disease or chronic schizophrenia, oral doses of 5, 7, and 9 mg produced a dose-related increase in plasma prolactin and a smaller increase in growth hormone [63]. Muscimol was also evaluated in patients with tardive dyskinesia at oral doses of 5–9 mg [64]. The theoretical 24.8 mg MUS yield from the IA fraction is therefore several-fold greater than the 5–9 mg doses used in these historical studies, although mushroom exposure and administration of purified muscimol are not directly equivalent.

These studies establish human pharmacological activity of purified muscimol in the single-digit milligram range, but they do not define a validated toxic-dose threshold. Therapeutic, neuroendocrine, sedative, and other neurological effects occurred within overlapping dose ranges and in small, selected clinical populations. Mushroom intoxication adds further uncertainty because the exposure contains IA as well as MUS, and the absorbed dose depends on the initial composition, fruiting-body mass, processing, conversion of IA to MUS, gastrointestinal absorption, and co-exposures. For these reasons, a single numerical “toxic dose” for *A. muscaria* or muscimol would imply a precision that the human evidence does not support.

Human toxicokinetic information is similarly incomplete. Clinical effects generally emerge within hours of ingestion, but their onset and duration vary with the preparation, amount consumed, food intake, individual susceptibility, and co-exposures [2,3,5,31,60,65,66].

Detection of IA or MUS in serum or urine can confirm exposure [17,49,51,52,67], yet an isolated concentration is difficult to interpret without a validated concentration–time relationship. A low result may reflect limited exposure, delayed sampling, rapid elimination, incomplete absorption, or analytical sensitivity rather than mild poisoning.

Modern studies designed specifically to establish a human toxicity threshold by controlled dose escalation would raise substantial ethical concerns. More informative evidence is likely to come from well-documented clinical cases in which the consumed material is chemically characterized and biological samples are collected at known times. Pairing these measurements with standardized clinical observations and documentation of co-exposures would allow concentration–time and dose–effect relationships to be assessed without treating historical experimental doses as safety limits.

### 7.6. Diagnostic Considerations

Diagnosis is usually based on exposure history, clinical findings, mushroom or product identification, and exclusion of other causes [2,3,5,60]. Diagnostic uncertainty is common because consumed material may be cooked, dried, fragmented, mixed with other species, or unavailable for analysis. Symptoms may overlap with sedative intoxication, anticholinergic delirium, hallucinogen exposure, alcohol intoxication, neurological disease, or acute psychiatric presentations.

Chemical confirmation by LC–MS/MS or a comparably validated method may identify ibotenic acid and muscimol in mushroom material, products, serum, urine, or gastric contents. Such testing is valuable in forensic, regulatory, or diagnostically uncertain cases but is not routinely available in all clinical settings.

Species identification and chemical analysis answer different questions. Taxonomic confirmation identifies the likely biological source, whereas quantitative chemical analysis characterizes the actual exposure. Both may be necessary when commercial products contain complex extracts or undeclared substances.

### 7.7. Clinical Management and Outcome

The published clinical literature describes management as primarily supportive [2,3,5,60]. Clinical priorities may include monitoring of consciousness and vital functions, prevention of aspiration, correction of fluid loss, and treatment of severe agitation or seizures. No specific antidote for ibotenic acid or muscimol poisoning has been established; detailed treatment protocols fall outside the scope of this chemistry- and analytical-toxicology-focused review.

Outcome is favorable in many reported cases, but severe intoxication can occur, particularly following high or uncertain doses, exposure to concentrated products, co-ingestion, or in vulnerable individuals. Case reports of severe outcomes establish clinical possibility but cannot provide reliable incidence estimates because reporting is selective and denominator data are usually unavailable.

### 7.8. Veterinary Relevance

Dogs and other animals may ingest fruiting bodies accidentally and develop vomiting, disorientation, ataxia, tremors, sedation, agitation, or seizures. Veterinary reports confirm that isoxazole-containing mushrooms can produce clinically important neurological poisoning in animals [68,69]. A 2026 veterinary case report described fatal *A. muscaria* poisoning in three Old Polish drakes and confirmed exposure by LC–MS/MS determination of ibotenic acid and muscimol in post-mortem specimens [68]. The report provides analytically supported evidence of severe avian intoxication, although its case-based design does not permit inference about toxic-dose thresholds or population-level veterinary risk.

The veterinary evidence is nevertheless heterogeneous. Exposure is often inferred from access to mushrooms and clinical signs, while quantitative chemical confirmation is uncommon. Species differences in metabolism and sensitivity also limit direct extrapolation between animals and humans.

Ecological claims should be separated from veterinary observations. Animal poisoning demonstrates biological activity after ingestion but does not prove that ibotenic acid or muscimol evolved as deterrents against fungivory.

Experimental evidence firmly establishes the excitatory pharmacology of ibotenic acid and the GABA-receptor agonism of muscimol, while clinical reports support the occurrence of mixed neurological manifestations after ingestion of isoxazole-containing *Amanita* species. By contrast, quantitative dose–response relationships, associations between biological concentrations and clinical severity, ecological functions, and prediction of symptoms from the IA/MUS ratio remain insufficiently established.

## 8. Environmental Regulation of the IA–MUS Profile: Current Evidence and Uncertainty

### 8.1. Evidence Boundary

Environmental regulation of fungal secondary metabolism is well documented in a broad range of fungi. Temperature, nutrient status, water availability, developmental signals, and biotic interactions can alter pathway expression or metabolite production [19,20,21,22,23,24,25,26]. This literature provides a reasonable basis for asking whether the ibotenic acid pathway is environmentally responsive, but it does not answer that question for *A. muscaria*.

For IA and MUS specifically, direct controlled evidence remains sparse. No study has yet established a causal relationship between an ecological change and altered ibotenic acid biosynthesis in intact *A. muscaria* fruiting bodies. Field observations are particularly difficult to interpret because environmental conditions covary with fruiting-body age, tissue composition, hydration, host association, and the interval between collection and analysis.

The identification of L-glutamate hydroxylation as the entry point to ibotenic acid biosynthesis provides a molecular basis for future regulatory studies [18]. It does not, however, show that environmental glutamate or nitrogen availability determines pathway output. For this reason, evidence from general fungal biology is treated here as mechanistic context rather than direct evidence for environmental control of IA or MUS production.

### 8.2. Candidate Environmental Variables

Several environmental variables are plausible candidates for further investigation, but none can currently be regarded as a demonstrated regulator of the IA–MUS profile in *A. muscaria*.

Temperature may influence fungal growth, enzyme activity, fruiting, and host-tree physiology, yet post-harvest heating cannot be used as evidence for temperature-dependent biosynthetic regulation in living tissue. Water availability presents a similar interpretative problem. Drought can affect fungal physiology, but it also changes tissue hydration; an apparent increase in concentration per gram of fresh material may therefore occur without any increase in metabolite production [70].

Nitrogen status deserves particular attention because glutamate is the known biosynthetic precursor of ibotenic acid [18]. Even so, precursor identity alone does not establish substrate limitation. Pathway expression, enzyme activity, intracellular allocation, and developmental state may be more important than environmental nitrogen supply.

Soil chemistry and metal exposure add another layer of complexity. *A. muscaria* is capable of accumulating metallic and metalloid elements [71,72], but metal accumulation and regulation of isoxazole biosynthesis are separate phenomena. Likewise, microbial interactions and host-tree condition may alter nutrient exchange or fungal physiology [73], yet no direct evidence currently links either process to IA or MUS production.

Taken together, these observations justify environmental variables as experimental candidates, not as established determinants of chemical phenotype. Their current evidence status and the main sources of uncertainty are summarized in Table 4.

Table 4 summarizes the present evidence for candidate environmental variables and the main uncertainties that limit causal interpretation.

Developmental stage, tissue selection, hydration, and post-harvest processing are treated separately in Section 4 and Section 5. They are important sources of variation and potential confounders, but they are not classified here as environmental determinants of biosynthesis.

## 9. Commercial Products, Public Health, and Regulatory Relevance

### 9.1. Changing Patterns of Exposure

Human exposure to *Amanita muscaria* is no longer restricted to accidental ingestion of wild fruiting bodies. Dried mushrooms, powders, extracts, capsules, gummies, chocolates, and liquid formulations are increasingly available through online and retail markets. These products may be promoted for psychoactive, wellness, or nootropic purposes, although their chemical composition and dose standardization are often uncertain [27,28,29,30,31,66,74].

Wild-mushroom ingestion and commercial-product exposure should be treated as distinct scenarios. In accidental foraging cases, the principal uncertainties include species identification, the quantity consumed, developmental stage, and preparation method. Commercial products introduce additional variables, including the origin of the raw material, extraction conditions, formulation, batch consistency, storage, and possible adulteration.

Recent product evidence is best interpreted as a series of time- and location-specific snapshots rather than as a representative survey of the wider market. In the Virginia investigation reported by Michienzi et al., six retail packages representing five brands were purchased near Charlottesville in October 2023 and analysed by qualitative LC–QToF-MS [28]. A separate study by Katragunta et al. examined 27 consumer products across several formulation types using a validated quantitative LC–QToF-MS method [55]. These datasets are informative for product composition and labelling, but neither supports prevalence estimates for all products marketed as containing *A. muscaria*. Product-level findings and their analytical limitations are summarized in Supplementary Table S4.

A product labelled as containing *A. muscaria* cannot be assumed to reproduce the chemical profile of a fresh fruiting body. Unless fungal identity has been independently verified, such products are treated here as commercial exposure matrices rather than as authenticated *A. muscaria* specimens. Processing may change the concentrations of ibotenic acid and muscimol, while formulated matrices may contain additional active or interfering substances.

### 9.2. Composition and Labelling Uncertainty

Product labels may identify a mushroom species, an extract mass, a proprietary blend, or a stated amount of muscimol. These declarations do not necessarily provide sufficient information for toxicological interpretation. An extract mass does not indicate the concentrations of individual active compounds, and a declared muscimol content does not exclude residual ibotenic acid or other constituents.

Product testing has revealed both label–content discordance and important analytical limitations. In the Virginia survey, six retail packages were screened by qualitative LC–QToF-MS; several contained undeclared psychoactive compounds, but IA, MUS, and muscarine were not included in the matching library and their presence could therefore not be determined [28]. In the quantitative survey by Katragunta et al., 27 consumer products were examined, including 14 gummies. Eleven of the gummies were labelled as containing Amanita extracts; MUS and muscarine were detected in these products, whereas IA was not detected [55]. The same study identified further label–content discrepancies, including a gummy labelled as containing no psilocybin in which psilocin and psilocybin were detected [55]. These findings support product-specific analytical verification rather than generalization to the wider market.

Three forms of label verification should be distinguished:

- Identity verification, determining which fungal or non-fungal ingredients are present;
- Quantitative verification, measuring the concentrations of ibotenic acid, muscimol, and other relevant compounds;
- Batch-consistency assessment, determining whether separately manufactured units have comparable composition.

A single qualitative detection result is insufficient to establish dose accuracy or product consistency.

### 9.3. Analytical Requirements for Commercial Products

Commercial formulations require matrix-specific methods. Gummies, chocolates, capsules, beverages, and tinctures may contain sugars, gelatin, fats, acids, flavours, colourants, botanical extracts, and preservatives. These constituents can alter extraction efficiency, chromatographic separation, and ionization during mass spectrometric analysis.

Methods developed for mushroom tissue should not be transferred directly to finished products without validation. Appropriate testing should include:

- Recovery of both ibotenic acid and muscimol from the finished matrix;
- Matrix-matched calibration;
- Assessment of ion suppression or enhancement;
- Homogeneity testing within individual products;
- Comparison between batches;
- Stability during storage;
- Screening for undeclared pharmacologically active compounds.

Targeted LC–MS/MS is appropriate when the expected analytes are defined. Broader screening may be required when adulteration or mislabelling is suspected. Multi-analyte workflows can include muscarine, psilocybin, psilocin, selected mushroom toxins, pharmaceuticals, or synthetic psychoactive substances, depending on the analytical and regulatory question.

The presence of a compound should not be interpreted as proof that it caused a reported adverse event. Exposure assessment requires quantitative results, information on the amount consumed, timing, co-ingestions, and relevant clinical findings.

### 9.4. Public Health Interpretation

Public health assessment should distinguish chemical hazard from exposure risk. Ibotenic acid and muscimol possess established neuropharmacological activity, but the risk associated with a particular product depends on the amount present, serving size, consumption pattern, co-exposures, and individual vulnerability.

Several characteristics of commercial products increase uncertainty:

1. The starting fungal material may not be authenticated.
2. Processing can alter the IA–MUS profile.
3. Active-compound concentrations may not be stated.
4. Labelled serving sizes may not correspond to analytically verified doses.
5. Individual units or batches may differ.
6. Additional psychoactive substances may be present.

These uncertainties make adverse events difficult to interpret when no product sample remains for analysis. Poison-centre and clinical reports should therefore record the exact product name, manufacturer, batch or lot number, package information, amount consumed, co-ingestions, symptom onset, and availability of remaining material.

Surveillance should also distinguish between exposure to wild mushrooms and manufactured products. The former primarily requires species identification and foraging education; the latter requires product testing, traceability, accurate labelling, and batch-level quality control. Surveillance reports should record product form, preparation method, estimated dose, analytical confirmation, co-exposures, and clinical outcome.

### 9.5. Regulatory Relevance

In the United States, the FDA stated on 18 December 2024 that *A. muscaria*, its extracts, muscimol, ibotenic acid, and muscarine are not authorized as ingredients in conventional food and do not meet the Generally Recognized as Safe (GRAS) standard for that use. This statement describes the U.S. regulatory position at that time and should not be generalized to other jurisdictions [29,30].

This regulatory material should be used to establish the relevance of product safety and analytical verification, not as evidence for fungal biosynthesis or environmental regulation. Regulatory documents address whether available safety information is adequate for a particular use; they do not establish how ecological stress affects the production of ibotenic acid.

### 9.6. Evidence Limitations

The available public health literature demonstrates that exposure to commercial mushroom products is an emerging safety concern. However, several limitations restrict interpretation:

- The market changes rapidly;
- Product formulations may change without corresponding changes to the brand name;
- Convenience samples do not establish the prevalence of adulteration across the entire market;
- Case reports may lack chemical analysis of the consumed product;
- Adverse-event surveillance cannot reliably estimate incidence without sales or exposure denominators;
- Analytically detected constituents cannot always be causally linked to reported symptoms.

Results from an individual product or batch do not characterize the wider market. Product names and label claims alone are insufficient to establish composition when analytical identity is relevant to safety, clinical interpretation, forensic investigation, or regulation.

### 9.7. Practical Reporting Implications

Commercial-product and clinical reports require a small set of additional identifiers beyond the general analytical framework described in Section 10.1. Product studies need manufacturer and batch information, label claims, matrix-specific validation, and quantitative IA and MUS results. Clinical reports are most useful when the amount consumed, timing, co-exposures, patient characteristics, treatment, and outcome are documented. Retention of remaining product material also allows subsequent chemical confirmation when the initial exposure assessment is uncertain.

## 10. Research Gaps and Priorities

### 10.1. Need for Standardized and Comparable Data

The principal limitation of the current literature is not the complete absence of chemical data, but the difficulty of comparing results obtained using different biological material, processing conditions, analytical procedures, and reporting formats. Studies vary in species authentication, developmental stage, anatomical tissue, storage history, extraction protocol, analytical platform, calibration strategy, and concentration units.

Comparable IA and MUS datasets require a common reporting framework [8,10,11,12,13,14,16,17,47,49,52,75].

**Figure 4:**
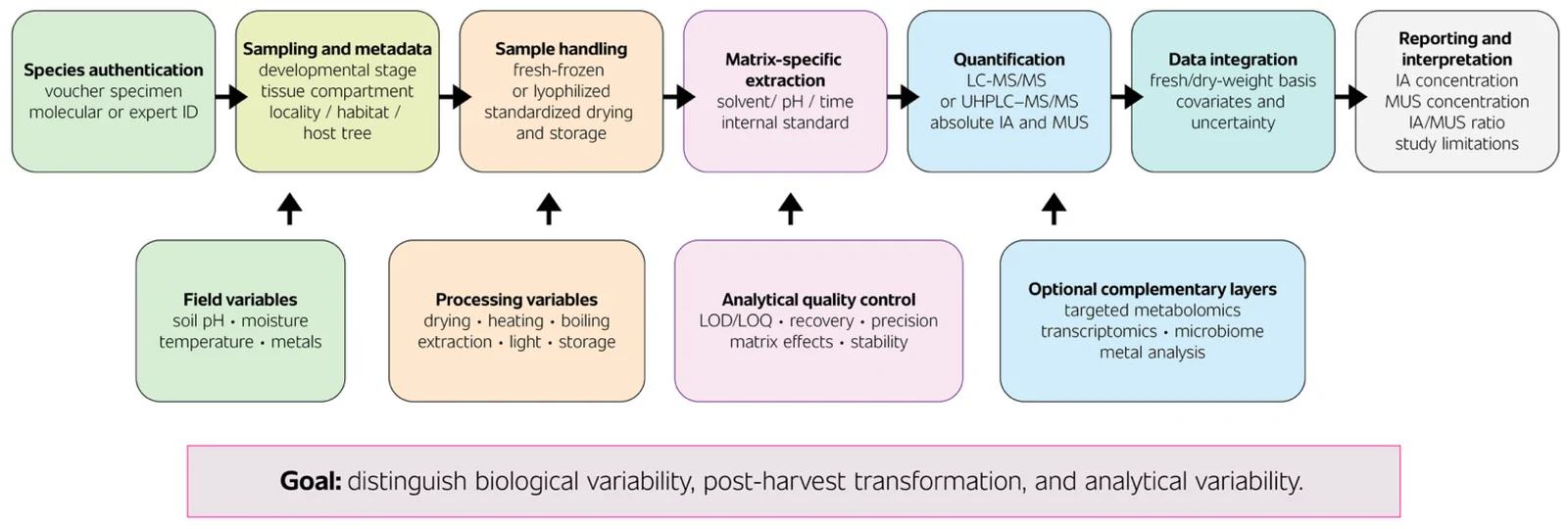
Recommended workflow for studies of ibotenic acid and muscimol variability. Reliable comparison requires authenticated biological material, standardized sampling, documented post-harvest handling, matrix-specific validation, and reporting of absolute IA and MUS concentrations together with the IA/MUS ratio and relevant sources of uncertainty.

The essential information falls into four domains: biological identity and sampling, developmental and anatomical context, post-harvest history, and analytical performance. Together, these cover species authentication and voucher information, biological replication, tissue and developmental stage, moisture and normalization basis, processing and storage, extraction, calibration, recovery, precision, matrix effects, LOD/LOQ, analyte stability, and absolute IA and MUS concentrations. The IA/MUS ratio provides an additional descriptor when the underlying concentrations are also reported. Figure 4 summarizes this workflow, while the study-level analytical considerations are discussed in Section 6.

### 10.2. Biological Distribution and Pathway Regulation

The evidence for biological variability is narrower than the number of published discussions might suggest. Taxonomic inference relies primarily on the multi-species survey by Su et al. [8], while developmental evidence is based mainly on one direct *A. muscaria* study [9] and observations in *A. subglobosa* [8]. Broader metabolomic and genomic studies add useful context [45,47], but they do not substitute for replicated IA- and MUS-specific measurements. The immediate need is therefore not simply for more observations, but for independent datasets using authenticated *A. muscaria* material, defined developmental stages, standardized anatomical sampling, and comparable quantitative methods.

Chemical analysis alone cannot determine whether observed differences reflect altered biosynthesis, tissue expansion, hydration, redistribution, degradation, or conversion to muscimol. Gene-expression analysis, enzyme assays, or stable-isotope tracing would provide stronger evidence of changes in pathway activity [18].

### 10.3. Controlled Evaluation of Post-Harvest Transformation

Post-harvest studies should use replicated portions of a common homogenized starting material and report temperature, duration, pH, moisture, light exposure, and storage conditions. A mass-balance design should quantify ibotenic acid, muscimol, relevant degradation products, and all applicable solid and liquid fractions to distinguish decarboxylation, leaching, degradation, water loss, and incomplete analytical recovery [10,11,15].

Results should remain specific to the tested procedure. Findings obtained under one drying, cooking, storage, or extraction protocol should not be generalized to other processing conditions without direct analytical confirmation.

### 10.4. Environmental Regulation as an Experimental Question

Environmental regulation remains one of the clearest unresolved questions in *A. muscaria* chemistry. Section 8 shows that temperature, water availability, nutrient status, soil chemistry, metals, and biotic interactions are plausible candidates, but none has yet been linked causally to IA or MUS production. Future work should therefore concentrate on a small number of mechanistically justified variables rather than expanding the list of possible environmental associations [18,19,20,21,22,23,24,25,26,70].

A convincing experiment would pair quantitative IA and MUS measurements with an independent indicator of pathway activity, such as gene expression, enzyme activity, or metabolic flux, while controlling developmental stage, tissue, hydration, and post-harvest handling. Ecological functions such as fungivore deterrence or microbial competition remain separate questions and would require their own experimental evidence.

### 10.5. Analytical Validation and Interlaboratory Performance

Future methodological work should prioritize comparability and robustness rather than the development of additional platform variants. Interlaboratory studies using shared reference materials and blinded samples should assess extraction recovery, calibration, precision, matrix effects, analyte stability, and agreement between analytical platforms [8,11,12,13,14,16,17,49,52].

Method selection should remain application-specific. Ecological studies require reproducibility and throughput, clinical and forensic analyses require high selectivity and low detection limits, and regulatory product testing requires matrix-specific validation and broad screening for undeclared compounds.

### 10.6. Linking Chemical Composition with Clinical Effects

Future clinical reports should combine confirmed species or product identity, estimated dose, preparation method, timed biological sampling, standardized symptom and outcome data, and quantitative analysis of any remaining material [2,3,5,17,27,28,31,49,52,60,66,74]. Such datasets would allow evaluation of whether absolute IA and MUS concentrations or their relative proportions are associated with clinical presentation.

Commercial-product studies should additionally report the manufacturer and batch because formulations may change over time. Until paired chemical–clinical data are available, the IA/MUS ratio should not be regarded as a validated predictor of symptom pattern or severity.

### 10.7. Prioritized Research Agenda

The principal evidence gaps, limitations of the available studies, and recommended study designs are summarized in Table 5.

## 11. Limitations of the Review

The main limitation of this review is the size and heterogeneity of the underlying evidence base. The literature combines analytical-method studies, small biological surveys, mechanistic experiments, case reports, poison-centre data, and regulatory documents. These sources answer different questions and cannot always be compared directly.

Evidence is particularly uneven for biological variability. Taxonomic distribution is informed largely by one recent multi-species dataset [8], while developmental and anatomical evidence for *A. muscaria* comes from a small number of studies [9,47]. A further limitation is that several foundational observations concerning *A. muscaria* development, tissue distribution, drying, storage, and early analytical determination derive from three publications by the same research group, all published in the same journal in 1993 [9,10,11]. Although these studies addressed distinct experimental questions, they do not constitute independent interlaboratory replication. Modern confirmation using replicated sampling and contemporary analytical methods would therefore strengthen these conclusions.

Environmental regulation remains more speculative: findings from other fungal systems establish biological plausibility but cannot demonstrate equivalent control of the ibotenic acid pathway in *A. muscaria*.

Methodological heterogeneity places a further limit on quantitative synthesis. Published studies differ in matrix, sample preparation, analytical platform, validation, and reporting units, while clinical reports rarely combine analysis of the consumed material with timed biological sampling and standardized outcome data. Commercial-product evidence is additionally time- and batch-dependent. These limitations are the reason the review emphasizes study-level context rather than numerical pooling across incompatible datasets.

## 12. Conclusions

Ibotenic acid and muscimol are structurally related fungal isoxazole metabolites with distinct pharmacological properties. Their chemical relationship is defined by the decarboxylation of ibotenic acid to muscimol, but the composition of Amanita material is influenced by more than this single transformation.

Direct evidence supports interspecific variation within *Amanita* section *Amanita* and shows that maturation and tissue selection can influence the measured IA–MUS profile, although these conclusions still depend on a small number of independent studies [8,9,47]. Processing-related changes during defined drying and heating conditions are supported separately by experimental data, while analytical interpretation remains strongly dependent on matrix-specific validation.

Sample maturity, tissue selection, hydration, storage, extraction, and concentration units can all affect the reported chemical profile, complicating attribution of differences to biological or geographic factors.

LC–MS/MS and UHPLC–MS/MS currently provide suitable platforms for confirmatory quantification of ibotenic acid and muscimol, particularly when high selectivity and sensitivity are required. Capillary electrophoresis and CE–MS approaches may provide complementary options for selected applications. No technique should be considered universally superior without reference to the matrix, validation parameters, throughput requirements, and intended use.

The IA/MUS ratio may be useful as a secondary descriptor of sample composition, but it should always be reported together with absolute concentrations. Its toxicological interpretation is limited by total dose, preparation method, matrix composition, co-exposures, toxicokinetics, and individual susceptibility. Current clinical evidence does not support using the ratio as an independent predictor of symptom pattern or severity.

Environmental regulation of ibotenic acid biosynthesis remains an unresolved question. General fungal-metabolism studies provide mechanistic plausibility, but direct controlled evidence in *A. muscaria* is insufficient. Temperature, water availability, nitrogen status, soil properties, metals, microbial interactions, and host-tree physiology remain candidate variables for experimental testing rather than established determinants of the IA–MUS profile.

Progress in this field depends on standardized biological sampling, transparent reporting of post-harvest conditions, validated quantitative methods, and clearer separation of direct evidence from mechanistic inference. Replicated developmental studies, controlled processing experiments, pathway-level analyses, interlaboratory comparisons, and paired chemical–clinical datasets would provide the evidence needed to define the sources and toxicological consequences of IA–MUS variability more reliably.

## Figures and Tables

**Table 1:** Principal compounds relevant to the chemistry and toxicology of *Amanita muscaria*. Evidence summarized from [1,6,7,15,34,35,38,39,41,42].

Compound	Chemical Relationship	Principal Established Pharmacology	Taxonomic Scope and Relevance to *A. muscaria*	Main Interpretative Limitation
Ibotenic acid	Isoxazole amino acid with a stereogenic α-carbon; natural product reported as racemic; precursor of muscimol	Agonist at NMDA and group I/II metabotropic glutamate receptors; NMDA-mediated experimental excitotoxicity	Directly documented in *A. muscaria*; also detected in several related species within section *Amanita*	Experimental pharmacology does not directly predict the clinical effects of ingested material
Muscimol	Achiral decarboxylation product of IA; ionizable, tautomeric isoxazole system	Ionotropic GABA-receptor agonist	Directly documented in *A. muscaria*; also detected in related species within section *Amanita*	Clinical effects depend on dose, matrix, IA content and co-exposures
Muscazone	Light-associated transformation product of ibotenic acid; mechanism incompletely resolved	Limited pharmacological relevance	Directly reported as a minor constituent or transformation product in *A. muscaria*	Quantitative and toxicological data remain limited
Muscarine	Chemically distinct cholinergic compound	Muscarinic receptor agonist	Present in *A. muscaria* at comparatively low levels; greater toxicological relevance occurs in other fungal taxa	Concentration and relevance differ markedly among fungal taxa

Evidence categories describe the strength of the claim within its stated taxonomic scope. Results from related *Amanita* species are not treated as direct evidence for *A. muscaria*. Where both *A. muscaria* and related-species data are available, they are identified separately.

**Table 2:** Primary evidence supporting biological variability in ibotenic acid (IA) and muscimol (MUS), with study-level information used to assign evidence categories.

Factor	Primary Evidence (No. of Studies)	Species/ Sample Size	Analytical Approach	Evidence Category	Interpretation and Principal Limitation
Species identity	2 targeted studies [8,44]	[8]: 84 samples representing 24 species of *Amanita* section *Amanita*. [44]: two species; three documented collections (*A. parvisychnopyramis*, 2; *A. flavomelleiceps*, 1); analytical replicate number not reported separately	UPLC–MS/MS [8]; LC–MS/MS [44]	Direct for the analysed species	Interspecific differences in IA/MUS occurrence are directly supported. The evidence is still dominated by one large regional survey [8], while ref. [44] provides a small independent extension. Analytical non-detection remains sample- and method-specific
Developmental stage	2 datasets [8,9]	[9]: *A. muscaria*, *n* = 62 fruiting bodies, examined across five maturation stages. [8]: *A. subglobosa*, developmental subset of the 84-sample survey; exact subset size not reported separately in the abstract.	Ion-pair RP-HPLC–UV [9]; UPLC–MS/MS [8]	Direct but limited; ref. [8] is direct for *A. subglobosa* but indirect for an *A. muscaria*-specific claim	In *A. muscaria*, maturation was associated with tissue-dependent changes while whole-fruiting-body concentrations remained comparatively stable [9]. *A. subglobosa* showed lower IA and MUS concentrations at maturity [8]. The two datasets do not establish a universal maturation trajectory.
Tissue compartment	2 studies [9,47]	[9]: *A. muscaria*, *n* = 62; cap, stipe, base, and whole fruiting body. [47]: *A. muscaria*, 22 fruiting bodies yielding 44 paired cap/stipe samples.	Ion-pair RP-HPLC–UV; ^1^H NMR metabolomic profiling	Direct but limited	Ref. [9] provides direct IA/MUS evidence of anatomical heterogeneity, while ref. [47] independently demonstrates broader chemical differences between caps and stipes. Modern replicated targeted mapping across all anatomical compartments remains lacking.
Individual and geographic variation	2 relevant datasets; no dedicated targeted geographic IA/MUS study [9,45]	[9]: *A. muscaria*, *n* = 62. [45]: *A. muscaria*, 24 Northern- and Southern-Hemisphere specimens/genomes.	Ion-pair RP-HPLC–UV [9]; genome sequencing and untargeted MS metabolomics [45]	Direct for specimen-level observations and genomic/metabolomic comparison; indirect for geographic IA/MUS concentration patterns	Specimen-level variability is supported by [9], although that study found no consistent effect of lone versus colonial growth or fruit-body size. Ref. [45] provides an intercontinental genomic and metabolomic comparison but does not establish equivalence or geographic differences in targeted IA/MUS concentrations. Robust geographic IA/MUS concentration patterns have not been established.

The number of studies refers to independent primary datasets relevant to the stated factor; reviews and general mechanistic sources are not counted. “Direct” refers to evidence for the species or material stated in the corresponding row. Findings from another *Amanita* species become indirect when extrapolated to an *A. muscaria*-specific claim. Hydration and normalization are treated separately in Section 4.5 as methodological modifiers rather than biological evidence categories. Genomic evidence for the *ibo* biosynthetic gene cluster is discussed separately in Section 3.4 and Figure 2 and is not counted here as direct evidence of quantitative IA/MUS production.

**Table 3:** Study-level comparison of representative analytical methods used for the determination of ibotenic acid (IA) and muscimol (MUS).

Species or Matrix (Reference)	Analytical Method	Sample Preparation/ Internal Standard	Calibration Range	LOD/ LOQ	Performance and Practical Relevance
*Amanita* mushrooms [16]	HPLC–UV with LC–ESI–MS/MS confirmation	Aqueous MeOH extraction; DNS-Cl derivatization followed by ethylation	MUS: 25–2500 ppm; IA: 40–2500 ppm	NR	Quantitative method with MS confirmation; two-step derivatization increases sample-preparation complexity
*Amanita* mushroom material [12]	HILIC–LC–MS/MS, MRM	H_2_O/MeOH (1:1); Oasis MAX SPE; acivicin as IS; no derivatization	IA and MUS: 10–500 μg/g	NR	Recovery 84.6–107%; *r* ≥ 0.99; chromatographic program ≤ 10 min. Direct quantitative analysis without derivatization
*A. muscaria*, *A. pantherina*, *A. citrina* [13]	CZE–UV	Ultrasound-assisted MeOH/phosphate-buffer extraction; no derivatization	IA and MUS: 2.5–7000 μg/mL	See original study	Recovery > 87%; intraday RSD 1.0%, interday RSD 2.5%. Wide quantitative range, but lower molecular specificity than MS-based confirmation
human urine [14]	CE–ESI–MS/MS	Minimal sample pretreatment; no derivatization	IA/MUS: low ng–μg/mL range	Nanomolar range	Migration-time RSD 0.93–1.60%; peak-area RSD 2.96–3.42%; *r*^2^ > 0.999. High sensitivity with limited pretreatment
human urine from *A. pantherina* poisoning [49]	GC–MS	Dowex 50W X8 cation exchange; ethyl-chloroformate derivatization; cycloserine as IS	Study-specific	Study-specific	Applied to urine from four intoxicated patients; robust confirmation but requires extraction and derivatization
human serum [51]	HILIC–LC–MS/MS, SRM	100 μL serum; Oasis MAX SPE; acivicin as IS; no derivatization	IA and MUS: 10–1000 ng/mL	LOD: IA 1.0 ng/mL; MUS 2.5 ng/mL	Recovery 87.9–103%; *r* ≥ 0.999. Demonstrates the need for matrix-specific re-optimization when moving from mushroom tissue to serum
human plasma [17]	LC–triple-quadrupole MS	ACN protein precipitation; bimolecular dansylation; isotope-labelled IS	IA: 1–500 ng/mL; MUS: 1–200 ng/mL	LOD: IA 0.3 ng/mL; MUS 0.1 ng/mL	IA recovery 90.7–111.4%, RSD 6.4–10.3%; MUS recovery 85.1–94.2%, RSD 5.0–8.9%. High sensitivity at the cost of derivatization
human urine [52]	^1^H NMR (1D-NOESY)	Simple urine preparation with buffered D_2_O/reference standard; no derivatization	Study-specific	μg/mL range	Rapid, structurally informative and capable of detecting other metabolites, but less sensitive than targeted tandem-MS methods. The method should be considered separately from LC–MS/MS and CE–MS
mushrooms, serum, urine, simulated gastric fluid [54]	Multi-class LC–MS/MS	Matrix-dependent extraction; serum cleanup; matrix-matched calibration	0.05–200 μg/L for the multi-analyte panel	LOQ: 0.01–0.2 mg/kg in mushrooms; 0.15–2.0 μg/L in biological matrices	Recoveries across the multi-toxin panel 73.0–110.3%, RSD ≤ 19.4%. Useful for broad toxicological screening rather than IA/MUS alone
mushroom tissue/extract [53]	Direct mushroom spray MS; AEPS–MS/MS	Direct tissue analysis or anion-exchange modified paper; no derivatization	IA: 5–40 μg/mL	AEPS LOD 1.3 μg/mL; LOQ 4.3 μg/mL	Accuracy −14.0 to +5.1%; precision 10.8–18.0%; screening in ~2–3 min. Faster, but less sensitive than the HPLC–MS/MS comparator (LOD 0.009 μg/mL)

Abbreviations: ACN, acetonitrile; AEPS–MS/MS, anion-exchange paper-spray tandem mass spectrometry; CZE, capillary zone electrophoresis; DNS-Cl, dansyl chloride; HILIC, hydrophilic interaction liquid chromatography; IA, ibotenic acid; IS, internal standard; LOD, limit of detection; LOQ, limit of quantification; MRM, multiple reaction monitoring; MUS, muscimol; NR, not reported in a directly comparable form; SPE, solid-phase extraction; RSD, relative standard deviation; SRM, selected reaction monitoring. Numerical performance values refer to the specific matrices, calibration procedures, and validation conditions used in the original studies and should not be interpreted as a direct cross-platform ranking. Species or matrix is stated explicitly because analytical performance in material from another *Amanita* species, a biological fluid, or a formulated product does not constitute evidence for biological variability in *A. muscaria*.

**Table 4:** Current evidence for candidate environmental influences on ibotenic acid and muscimol variability in *Amanita muscaria*. Evidence and mechanistic context summarized from [8,18,19,20,21,22,23,24,25,26,45,47,70,71,72,73].

Factor	Direct Evidence for Effects on IA/MUS in *A. muscaria*	Current Interpretation	Principal Limitation or Confounder
Ecological temperature	None or insufficient	Biologically plausible influence on fungal physiology; no demonstrated effect on IA or MUS production	Developmental stage, season, hydration, host physiology, and confusion with post-harvest thermal effects
Drought and water availability	None	Water status may affect fungal physiology, but concentration changes can also arise from altered tissue hydration	Fresh-weight concentration is strongly influenced by water content, maturity, and tissue expansion
Nitrogen availability	None	Mechanistically relevant because glutamate is a biosynthetic precursor, but pathway regulation by environmental nitrogen has not been demonstrated	Precursor availability does not establish rate limitation; pathway expression and developmental state are uncontrolled
Soil pH and chemistry	None	Potential indirect influence through nutrient availability, host physiology, or the associated microbiome	Multiple soil variables covary in field samples, preventing attribution to a single chemical factor
Heavy metals	Accumulation demonstrated; pathway regulation not demonstrated	Metal accumulation is established in *A. muscaria*, but its relationship to IA or MUS biosynthesis is unknown	Element accumulation and isoxazole production may vary independently and should not be treated as evidence of a shared mechanism
Microbial interactions	None	Ecologically plausible source of metabolic modulation, without IA- or MUS-specific evidence	Microbial composition, tissue condition, developmental stage, and local chemistry are difficult to separate in field material
Host-tree stress	None	Host condition may alter ectomycorrhizal resource exchange, but an effect on isoxazole production has not been demonstrated	Host physiology, fungal development, soil conditions, and seasonal effects are strongly interdependent

Unless explicitly identified as *A. muscaria*-specific, supporting studies in this table provide indirect mechanistic context from broader fungal or ectomycorrhizal research. None is treated as direct evidence that the corresponding environmental variable regulates IA or MUS biosynthesis in *A. muscaria*.

**Table 5:** Main evidence gaps and recommended study designs.

Research Gap	Why It Matters	Proposed Study Design	Expected Contribution
Lack of standardized reporting	Published concentrations are difficult to compare	Minimum reporting checklist and shared units	Improved cross-study comparability
Limited developmental data for *A. muscaria*	Evidence from related species cannot be directly extrapolated	Replicated developmental series with fresh- and dry-weight analysis	Definition of maturation-related variation
Poor tissue-specific mapping	Whole-fruiting-body analysis may conceal anatomical differences	Separate cap, gill, stipe, and surface-tissue quantification	Identification of tissue distribution
Incomplete processing mass balance	Degradation, conversion, leaching, and water loss are often conflated	Controlled processing with analysis of all fractions	Mechanistic interpretation of post-harvest change
Unverified environmental regulation	Support is largely derived from general fungal biology	Controlled exposure with chemical and pathway measurements	Test of causal environmental effects
Limited method comparability	Platform performance is reported in different matrices and formats	Interlaboratory comparison using common reference samples	Assessment of reproducibility and transferability
Weak chemical–clinical correlation	Most cases lack quantitative analysis of ingested material	Paired product, biological-fluid, and standardized clinical data	Evaluation of dose and IA–MUS relationships
Variable commercial products	Labels may not reflect identity or dose	Batch-based, matrix-validated multi-analyte surveillance	Improved regulatory and exposure assessment

## Data Availability

No new data were created or analyzed in this study. Data sharing is not applicable to this article.

## Supplementary Materials

The following supporting information can be downloaded at https://www.mdpi.com/article/10.3390/molecules31183232/s1, Table S1, Comparison of the scope and methodological emphasis of selected previous reviews relevant to *Amanita muscaria*, ibotenic acid, and muscimol; Table S2, Reproducible database-specific literature-search strategies; Table S3, Claim-level evidence classification of primary studies contributing to the review; and Table S4, Commercial-product evidence relevant to composition, labelling, and analytical interpretation of products marketed as containing *Amanita muscaria* or other mushroom nootropics.

## Abbreviations

The following abbreviations are used in this manuscript:

CE	Capillary electrophoresis
CE–ESI–MS/MS	Capillary electrophoresis–electrospray ionization tandem mass spectrometry
CNS	Central nervous system
FDA	U.S. Food and Drug Administration
GABA	γ-Aminobutyric acid
GC/MS	Gas chromatography–mass spectrometry
HILIC	Hydrophilic interaction liquid chromatography
IA	Ibotenic acid
IA/MUS ratio	Ibotenic acid–muscimol ratio
LC–MS/MS	Liquid chromatography–tandem mass spectrometry
LOD	Limit of detection
LOQ	Limit of quantification
MUS	Muscimol
NMDA	N-methyl-D-aspartate
