# Supplementary material for: Ibotenic Acid and Muscimol in Amanita muscaria: Chemistry, Sources of Variability, Analytical Determination, and Toxicological Significance
Source: Molecules. 2026 Sep 13;31(18):3232. doi: 10.3390/molecules31183232 (PMC13609693; doi:10.3390/molecules31183232)
Supplement: Supplementary file 1 [file molecules-31-03232-s001.zip › molecules-4459348-supplementary.pdf]

Supplementary Material

Ibotenic Acid and Muscimol in *Amanita muscaria*: Chemistry, Sources of Variability, Analytical Determination, and Toxicological Significance

Andrzej Günther, Barbara Bednarczyk-Cwynar, and Michał Tomczyk

Reference numbers correspond to the main manuscript.
NR, not reported in a directly comparable form.
Evidence classifications in Table S3 are claim-specific, as defined in Section 2 of the main manuscript.

Table S1. Comparison of the scope and methodological emphasis of selected previous reviews relevant to Amanita muscaria, ibotenic acid, and muscimol.

Review	Main emphasis	IA/MUS chemistry	Biological / post-harvest variability	Analytical methods	Clinical / public-health context	Distinctive limitation relative to the present review
Michelot and Melendez-Howell [4] (2003)	Broad synthesis of chemistry, biology, toxicology, and ethnomycology.	Foundational discussion of IA, MUS, muscarine, and related chemistry.	Describes biological and processing context, but predates several later targeted datasets.	Covers methods available at the time.	Extensive toxicological and ethnomycological perspective.	Predates the ibo biosynthetic pathway, modern LC-MS/MS validation literature, current commercial-product evidence, and the claim-level evidence framework used here.
Stebelska [6] (2013)	Fungal hallucinogens, with emphasis on analytical methods and biological activity.	Detailed treatment of IA and MUS among fungal hallucinogens.	Variability is not organized as a separate evidence domain.	Strong analytical-method perspective for the literature then available.	Summarizes biological activity and toxicological relevance.	Does not explicitly separate biological variability from post-harvest transformation or classify evidence at the level of individual claims.
Voynova et al. [1] (2020)	Toxicological, pharmacological, and potential biomedical relevance of A. muscaria.	Summarizes major active constituents and their pharmacology.	Discusses composition and preparation in a broader biomedical narrative.	Analytical methodology is not the principal organizing framework.	Major focus on toxicology, pharmacology, and proposed biomedical relevance.	Less emphasis on study-level analytical validation, matrix-specific comparability, and explicit evidence-tiering.
Ordak [31] (2026)	A. muscaria in the evolving novel psychoactive-substance landscape.	Chemistry considered mainly in relation to exposure and toxicity.	Processing and variability discussed in the context of use and risk.	Analytical issues are secondary to clinical and public-health interpretation.	Strong emphasis on use patterns, clinical toxicity, and public-health concerns.	Not centered on chemical comparability, processing mass balance, or claim-level classification of direct versus indirect evidence.
Present review	Critical synthesis of chemistry and biosynthesis, biological variability, post-harvest transformation, analytical determination, toxicology, and commercial products.	Integrates structural chemistry, IA-to-MUS conversion, and the ibo biosynthetic gene cluster.	Separates biological sources of variation from processing, storage, extraction, and analytical effects.	Compares methods using study-level performance data and matrix-specific validation rather than platform descriptions alone.	Links clinical and public-health interpretation to chemical identity, dose, product matrix, and analytical confirmation.	Designed to expose the strength and limitations of the underlying evidence and to provide a reporting framework for future IA/MUS studies.

IA, ibotenic acid; MUS, muscimol. The table is intended to define the methodological novelty of the present synthesis rather than to rank previous reviews by quality.

Table S2. Reproducible database-specific literature-search strategies.

Search strings are organized into thematic blocks to improve specificity and reproducibility. The same thematic concepts were translated into the database-specific syntax shown below. Search coverage and the final update date are reported in Section 2 of the main manuscript.

Source	Search block	Advanced search query	Implementation notes
PubMed	A - Chemistry and biosynthesis	("Amanita muscaria"[Title/Abstract] OR "fly agaric"[Title/Abstract] OR "Amanita section Amanita"[Title/Abstract]) AND ("ibotenic acid"[Title/Abstract] OR muscimol[Title/Abstract] OR muscazone[Title/Abstract] OR muscarine[Title/Abstract]) AND (chemistry[Title/Abstract] OR structure[Title/Abstract] OR biosynth*[Title/Abstract] OR "biosynthetic gene cluster"[Title/Abstract] OR iboH[Title/Abstract] OR iboD[Title/Abstract] OR phylogenom*[Title/Abstract])	Title/Abstract field searches. No study-design filter. Each thematic block is run separately.
PubMed	B - Biological and post-harvest variability	("Amanita muscaria"[Title/Abstract] OR "Amanita section Amanita"[Title/Abstract]) AND ("ibotenic acid"[Title/Abstract] OR muscimol[Title/Abstract]) AND (development*[Title/Abstract] OR maturation[Title/Abstract] OR tissue[Title/Abstract] OR cap[Title/Abstract] OR stipe[Title/Abstract] OR geographic[Title/Abstract] OR drying[Title/Abstract] OR heating[Title/Abstract] OR cooking[Title/Abstract] OR storage[Title/Abstract] OR extraction[Title/Abstract] OR stability[Title/Abstract])	
PubMed	C - Analytical methods	("ibotenic acid"[Title/Abstract] OR muscimol[Title/Abstract]) AND ("LC-MS"[Title/Abstract] OR "LC-MS/MS"[Title/Abstract] OR "UHPLC-MS/MS"[Title/Abstract] OR "UPLC-MS/MS"[Title/Abstract] OR HILIC[Title/Abstract] OR "capillary electrophoresis"[Title/Abstract] OR "CE-ESI-MS/MS"[Title/Abstract] OR NMR[Title/Abstract] OR "paper spray"[Title/Abstract] OR "ambient ionization"[Title/Abstract] OR validation[Title/Abstract])	
PubMed	D1 - Clinical and veterinary poisoning	("Amanita muscaria"[Title/Abstract] OR "fly agaric"[Title/Abstract] OR "Amanita pantherina"[Title/Abstract]) AND (poison*[Title/Abstract] OR toxic*[Title/Abstract] OR intoxicat*[Title/Abstract] OR clinical[Title/Abstract] OR case[Title/Abstract] OR patient*[Title/Abstract] OR veterinary[Title/Abstract])	
PubMed	D2 - Commercial products	("Amanita muscaria"[Title/Abstract] OR "fly agaric"[Title/Abstract]) AND (gumm*[Title/Abstract] OR chocolate*[Title/Abstract] OR supplement*[Title/Abstract] OR "commercial product"[Title/Abstract] OR "commercial products"[Title/Abstract] OR "consumer product"[Title/Abstract] OR "consumer products"[Title/Abstract] OR adulterat*[Title/Abstract] OR label*[Title/Abstract])	
PubMed	D3 - Historical human muscimol pharmacology	muscimol[Title/Abstract] AND (human*[Title/Abstract] OR patient*[Title/Abstract] OR clinical[Title/Abstract] OR schizophrenia[Title/Abstract] OR Huntington*[Title/Abstract] OR prolactin[Title/Abstract] OR "growth hormone"[Title/Abstract] OR dyskinesia[Title/Abstract])	
Scopus	A - Chemistry and biosynthesis	TITLE-ABS-KEY(("Amanita muscaria" OR "fly agaric" OR "Amanita section Amanita") AND ("ibotenic acid" OR muscimol OR muscazone OR muscarine) AND (chemistry OR structure OR biosynth* OR "biosynthetic gene cluster" OR iboH OR iboD OR phylogenom*))	Advanced TITLE-ABS-KEY searches. No study-design filter. Each thematic block is run separately.
Scopus	B - Biological and post-harvest variability	TITLE-ABS-KEY(("Amanita muscaria" OR "Amanita section Amanita") AND ("ibotenic acid" OR muscimol) AND (development* OR maturation OR tissue OR cap OR stipe OR geographic OR drying OR heating OR cooking OR storage OR extraction OR stability))	
Scopus	C - Analytical methods	TITLE-ABS-KEY(("ibotenic acid" OR muscimol) AND ("LC-MS" OR "LC-MS/MS" OR "UHPLC-MS/MS" OR "UPLC-MS/MS" OR HILIC OR "capillary electrophoresis" OR "CE-ESI-MS/MS" OR NMR OR "paper spray" OR "ambient ionization" OR validation))	
Scopus	D1 - Clinical and veterinary poisoning	TITLE-ABS-KEY(("Amanita muscaria" OR "fly agaric" OR "Amanita pantherina") AND (poison* OR toxic* OR intoxicat* OR clinical OR case OR patient* OR veterinary))	
Scopus	D2 - Commercial products	TITLE-ABS-KEY(("Amanita muscaria" OR "fly agaric") AND (gumm* OR chocolate* OR supplement* OR "commercial product" OR "commercial products" OR "consumer product" OR "consumer products" OR adulterat* OR label*))	
Scopus	D3 - Historical human muscimol pharmacology	TITLE-ABS-KEY(muscimol AND (human* OR patient* OR clinical OR schizophrenia OR Huntington* OR prolactin OR "growth hormone" OR dyskinesia))	
Web of Science Core Collection	A - Chemistry and biosynthesis	TS=(("Amanita muscaria" OR "fly agaric" OR "Amanita section Amanita") AND ("ibotenic acid" OR muscimol OR muscazone OR muscarine) AND (chemistry OR structure OR biosynth* OR "biosynthetic gene cluster" OR iboH OR iboD OR phylogenom*))	Advanced Topic (TS) searches. No study-design filter. Each thematic block is run separately.
Web of Science Core Collection	B - Biological and post-harvest variability	TS=(("Amanita muscaria" OR "Amanita section Amanita") AND ("ibotenic acid" OR muscimol) AND (development* OR maturation OR tissue OR cap OR stipe OR geographic OR drying OR heating OR cooking OR storage OR extraction OR stability))	
Web of Science Core Collection	C - Analytical methods	TS=(("ibotenic acid" OR muscimol) AND ("LC-MS" OR "LC-MS/MS" OR "UHPLC-MS/MS" OR "UPLC-MS/MS" OR HILIC OR "capillary electrophoresis" OR "CE-ESI-MS/MS" OR NMR OR "paper spray" OR "ambient ionization" OR validation))	
Web of Science Core Collection	D1 - Clinical and veterinary poisoning	TS=(("Amanita muscaria" OR "fly agaric" OR "Amanita pantherina") AND (poison* OR toxic* OR intoxicat* OR clinical OR case OR patient* OR veterinary))	
Web of Science Core Collection	D2 - Commercial products	TS=(("Amanita muscaria" OR "fly agaric") AND (gumm* OR chocolate* OR supplement* OR "commercial product" OR "commercial products" OR "consumer product" OR "consumer products" OR adulterat* OR label*))	
Web of Science Core Collection	D3 - Historical human muscimol pharmacology	TS=(muscimol AND (human* OR patient* OR clinical OR schizophrenia OR Huntington* OR prolactin OR "growth hormone" OR dyskinesia))	
Google Scholar	Focused supplementary searches	1. "Amanita muscaria" "ibotenic acid" muscimol<br>2. "Amanita muscaria" biosynthesis ibo<br>3. "Amanita muscaria" development tissue cap stipe "ibotenic acid" muscimol<br>4. "Amanita muscaria" drying storage cooking "ibotenic acid" muscimol<br>5. "ibotenic acid" muscimol "LC-MS/MS"<br>6. "ibotenic acid" muscimol "UHPLC-MS/MS"<br>7. "ibotenic acid" muscimol "UPLC-MS/MS"<br>8. "Amanita muscaria" poisoning clinical muscimol<br>9. "Amanita muscaria" gummies "commercial products"<br>10. muscimol human schizophrenia Huntington prolactin dyskinesia<br>11. "fungal secondary metabolism" environmental stress development	Focused topic-specific searches were used because long Boolean strings are not handled consistently by Google Scholar. These searches supplement the structured database searches and support identification of additional primary studies, reviews, public-health material, and citation leads.

Source	Search block	Advanced search query	Implementation notes
CrossRef, publisher websites, and reference lists	Targeted verification and citation searching	Targeted searches by article title, DOI, author name, and backward/forward citation chaining from relevant publications.	Used for bibliographic verification, publisher-level checking, and supplementary backward/forward citation searching. This route is not treated as an independent primary database.

Eligibility and evidence-screening rules

Studies were eligible when they addressed at least one of the following domains: identification or quantification of ibotenic acid and/or muscimol; biosynthesis or chemical transformation; taxonomic, developmental, tissue-specific, or processing-related variability; analytical methodology; pharmacological or toxicological effects; clinical or veterinary exposure; or the composition and safety of commercial products. Priority was given to peer-reviewed studies with clearly identified biological material, quantitative analytical data, validated methods, or direct toxicological relevance. Regulatory and public-health documents were included when they contained current exposure or product-safety information not available in the peer-reviewed literature. Broader fungal secondary-metabolism and environmental-stress literature was retained only as mechanistic context and was not treated as direct evidence for stress-dependent IA/MUS regulation in A. muscaria. No quantitative meta-analysis was attempted because the evidence base is heterogeneous in biological material, analytical platform, study design, and outcome reporting.

Search-string nomenclature note: both "UHPLC-MS/MS" and "UPLC-MS/MS" were included because the literature uses both designations; the original Su et al. comparative study [8] uses UPLC-MS/MS.

Table S3. Claim-level evidence classification of primary studies contributing to the review.

Evidence categories are assigned to the specific claim supported by each row. A study may be direct evidence for its own species or matrix but indirect when extrapolated to A. muscaria or to a different outcome. Reviews and general background sources are not counted as independent experimental datasets.

Ref.	Study / species or matrix	Study size	Analytical / experimental approach	Principal finding / claim supported	Evidence category	Main limitation	Review section(s)
[2]	Stoeva-Grigorova et al. (2025); human A. muscaria poisoning	2 cases plus literature review	Clinical case documentation	Documents acute A. muscaria toxicity in an elderly couple after home preparation.	Direct clinical evidence	Very small case series; limited ability to relate symptoms to a quantified IA/MUS dose.	7, 9
[3]	Satora et al. (2005); human A. muscaria poisoning	Single case report	Clinical case documentation	Supports the clinical description of fly-agaric poisoning and its neurological manifestations.	Direct clinical evidence	Single case; incomplete chemical characterization of the ingested material.	7
[5]	Meisel et al. (2022); severe human A. muscaria poisoning	2 cases	Clinical case documentation	Shows that severe outcomes, including fatality, can occur after A. muscaria exposure.	Direct clinical evidence	Small case number; dose and chemical composition are uncertain.	7
[8]	Su et al. (2023); Amanita section Amanita, China	84 specimens; 24 species	UPLC-MS/MS targeted quantification	IA and MUS detected in 10 of 24 species; establishes interspecific chemical variation within a common analytical framework.	Direct for the analysed species	Single large regional survey; does not establish a fixed chemical phenotype for every population or species.	3.4, 4.1
[8]	Su et al. (2023); developmental subset of A. subglobosa	Subset of the 84-specimen survey; exact subset size NR separately	UPLC-MS/MS targeted quantification	Lower IA and MUS concentrations were reported in mature than in less-developed A. subglobosa specimens.	Direct for A. subglobosa; indirect for A. muscaria	Species-specific developmental pattern cannot be assumed to apply to A. muscaria.	4.2
[9]	Tsunoda et al. (1993); A. muscaria development and tissues	n = 62; cap, stipe, base, and whole fruiting body	Ion-pair RP-HPLC-UV	Mean whole-fruiting-body concentrations were 343 ppm IA and 22 ppm MUS; tissue-specific changes occurred during maturation while whole-body concentrations were comparatively stable. Substantial specimen-level variation was also reported, without a consistent effect of lone versus colonial growth or fruit-body size.	Direct biological evidence	Older single dataset; anatomical and developmental findings need modern replicated confirmation.	4.2, 4.3, 4.4, 7.5
[10]	Tsunoda et al. (1993); A. muscaria drying, storage, and cooking	NR in a directly comparable summary form	Controlled post-harvest experiments with chromatographic IA/MUS analysis	Drying decreased IA and increased MUS; storage effects were condition-specific; cooking altered the measured profile through transformation and/or leaching.	Direct processing evidence	Experimental conditions are specific; modern mass-balance and moisture-normalized designs would improve interpretation.	5

Ref.	Study / species or matrix	Study size	Analytical / experimental approach	Principal finding / claim supported	Evidence category	Main limitation	Review section(s)
[11]	Tsunoda et al. (1993); A. muscaria and analytical survey of edible mushrooms	NR	Simultaneous chromatographic analysis of IA and MUS	Provides an early targeted analytical basis for IA/MUS determination and comparison among mushroom samples.	Direct analytical evidence	Older methodology and limited modern validation descriptors.	5, 6
[12]	Gonmori et al. (2012); Amanita mushroom material	NR	HILIC-LC-MS/MS; water/methanol extraction; Oasis MAX cleanup; acivicin internal standard; no derivatization	Demonstrated sensitive simultaneous IA/MUS analysis in mushroom matrix; reported 10-500 µg/g calibration and 84.6-107% recovery.	Direct analytical evidence	Performance is matrix- and method-specific; not all validation metrics are directly comparable with later studies.	6.2
[13]	Poliwoda et al. (2014); Amanitaceae mushrooms	NR	Capillary zone electrophoresis with UV detection	Demonstrated CE determination of IA and MUS; broad calibration range (2.5-7000 µg/mL) and recovery >87%.	Direct analytical evidence	UV detection is less structurally selective than tandem-MS confirmation; matrix-specific sensitivity differs from MS methods.	6.4
[14]	Ginterová et al. (2014); spiked human urine	Spiked matrix study	CE-ESI-MS/MS	Reported low detection limits for IA, MUS, and muscarine with high calibration linearity and acceptable migration/peak-area precision.	Direct analytical evidence for spiked urine	Validation in fortified urine is not equivalent to a patient-series clinical validation.	6.4, 6.5
[15]	Dushkov et al. (2023); A. muscaria hydroalcoholic extract and lung-cell models	Study-specific extract and in vitro cell panel	Targeted chemical analysis plus in vitro cytotoxicity testing	Characterized IA/MUS in a defined hydroalcoholic extract and examined cytotoxicity in vitro.	Direct for the tested extract and cell system	A single prepared extract does not represent fresh mushrooms or the wider commercial market; in vitro effects do not define human dose-response.	5, 6, 7
[16]	Tsujikawa et al. (2007); A. muscaria / A. pantherina mushroom samples	NR	HPLC and LC-MS/MS with derivatization	Established chromatographic and tandem-MS determination of IA and MUS in Amanita mushroom matrices.	Direct analytical evidence	Derivatization adds sample-preparation complexity; validation conditions are study-specific.	6.2, 6.3
[17]	Xu et al. (2020); plasma	Validation study	LC-triple-quadrupole MS with bimolecular dansylation	Calibration: IA 1-500 ng/mL; MUS 1-200 ng/mL. LODs 0.3 and 0.1 ng/mL; recovery 90.7-111.4% and 85.1-94.2%, respectively.	Direct analytical evidence	Derivatization improves sensitivity but adds preparation steps; plasma validation does not transfer automatically to mushroom matrices.	6.5
[18]	Obermaier and Müller (2020); A. muscaria biosynthetic pathway	Genomic/bioinformatic study plus recombinant-enzyme experiment	Gene-cluster identification and biochemical characterization of IboH	Identified the seven-gene ibo cluster and experimentally established IboH-catalysed L-glutamate hydroxylation as the first committed step.	Direct for ibo cluster / IboH step	Most downstream enzyme functions remain proposed rather than individually demonstrated with purified enzymes.	3.2, 3.3
[28]	Michienzi et al. (2024); retail mushroom/nootropic gummies, Virginia	6 packages; 5 brands	Qualitative LC-QToF-MS library matching	Detected undeclared psychoactive/other compounds in several retail products and demonstrated label-composition uncertainty.	Direct product evidence	Qualitative screen; IA, MUS, and muscarine were absent from the matching library and therefore could not be assessed by this analysis.	9.1-9.6
[33]	Brehm et al. (1998); ibotenic acid	Crystallographic / computational study	X-ray crystallography and quantum-chemical calculations	Established the zwitterionic monohydrate structure of crystalline ibotenic acid and supports explicit treatment of its stereochemistry/ionization.	Direct structural evidence	Solid-state structure does not by itself define the full solution speciation across pH.	3.1
[36]	Brehm et al. (1997); muscimol	Crystallographic / computational study	X-ray crystallography and quantum-chemical ab initio calculations	Established structural features of the muscimol zwitterion and supports explicit treatment of alternative solution protonation/tautomeric representations.	Direct structural evidence	Solid-state structure should not be equated with a single solution tautomer/protonation state.	3.1
[37]	Krogsgaard-Larsen et al. (1981); muscimol	Structure-activity / physicochemical study	Medicinal-chemistry and acid-base characterization	Supports classical pKa values of approximately 4.8 and 8.4 and the relationship of muscimol to GABAergic pharmacology.	Direct physicochemical / pharmacological evidence	Older study; not designed as an analytical validation or toxicokinetic study.	3.1, 7.1
[43]	Su et al. (2026); phylogenomics of Amanita section Amanita	25 genomes; 134 ibo homologues; 21 species with ibo genes	Comparative phylogenomics using the seven A. muscaria ibo genes as queries	All seven ibo genes recovered from 17 species and partial sets from four additional species; supports a broader evolutionary distribution of biosynthetic capacity.	Direct genomic evidence	Genomic presence establishes biosynthetic potential, not pathway expression or metabolite abundance.	3.2, 3.4
[44]	Su et al. (2025); A. parvisychnopyramis and A. flavomelleiceps	Taxonomic study; dried analysed specimens	Morphology, multilocus phylogeny, and targeted toxin analysis	IA and MUS detected in A. parvisychnopyramis; neither produced a corresponding signal in the analysed A. flavomelleiceps material.	Direct for the analysed species/material	Negative analytical result is condition-specific and cannot establish biological inability to produce the metabolites.	3.4
[45]	Nickles et al. (2026); European and South African A. muscaria	24 Northern- and Southern-Hemisphere specimens/genomes	Genome sequencing and untargeted MS metabolomics	Broad conservation of biosynthetic gene clusters and molecular families; only 13 of 273 molecular families were unique to South African material.	Direct genomic/metabolomic comparison; indirect for targeted IA/MUS geography	Untargeted conservation does not demonstrate equivalent IA/MUS concentrations across populations.	3.4, 4.4
[47]	Deja et al. (2014); A. muscaria cap versus stipe	22 fruiting bodies; 44 paired cap/stipe samples	1H NMR metabolomic profiling	Demonstrated broader low-molecular-weight chemical differences between cap and stipe.	Direct for broader tissue heterogeneity; indirect for targeted IA/MUS distribution	IA and MUS were not the sole targeted endpoints; cannot substitute for replicated targeted anatomical mapping.	4.3, 4.4

Ref.	Study / species or matrix	Study size	Analytical / experimental approach	Principal finding / claim supported	Evidence category	Main limitation	Review section(s)
[48]	Geml et al. (2008); A. muscaria phylogeography	NR here	Molecular phylogeography	Demonstrates substantial population structure in A. muscaria and provides geographic context for chemical sampling.	Direct phylogeographic evidence; indirect for IA/MUS chemistry	Genetic structure alone does not establish geographic IA/MUS concentration patterns.	4.4
[49]	Stříbrný et al. (2012); urine from A. pantherina-intoxicated patients	4 patients	Strong-cation-exchange isolation; ethyl-chloroformate derivatization; GC-MS; cycloserine internal standard	Quantified IA and MUS in clinical urine; calibration 1-15 μg/mL and LOD 1 μg/mL for both compounds.	Direct clinical-analytical evidence for A. pantherina	Small clinical series and different source species; cannot define A. muscaria toxicokinetics.	6.5, 7.5
[50]	Gennaro et al. (1997); A. muscaria	NR	Ion-interaction HPLC	Provides an early targeted chromatographic method for IA and MUS in A. muscaria.	Direct analytical evidence	Older chromatographic/detection framework; limited direct comparability with modern tandem-MS validation.	6.2
[51]	Hasegawa et al. (2013); human serum	Validation / application study	HILIC-LC-MS/MS; 100 μL serum; Oasis MAX cleanup; acivicin internal standard	Calibration 10-1000 ng/mL; LOD 1.0 ng/mL IA and 2.5 ng/mL MUS; recovery 87.9-103%.	Direct analytical evidence	Clinical application and matrix are distinct from mushroom assays; study-specific validation.	6.5
[52]	Deja et al. (2014); human urine	NR	1H NMR using a 1D-NOESY pulse sequence	Demonstrated rapid NMR determination of IA and MUS in urine without chromatographic separation.	Direct analytical evidence	Sensitivity/performance metrics are not directly comparable with targeted tandem-MS methods.	6.5
[53]	Luo et al. (2025); mushroom tissue / extract	Method-development study	Direct mushroom-spray MS and anion-exchange paper-spray tandem MS (AEPS-MS/MS)	Rapid tissue screening in about 2-3 min; IA range 5-40 μg/mL, LOD 1.3 μg/mL, LOQ 4.3 μg/mL; accuracy -14.0% to +5.1%, precision 10.8-18.0%.	Direct analytical evidence	Faster but less sensitive than the HPLC-MS/MS comparator used in the same study (LOD 0.009 μg/mL).	6.6
[54]	Lu et al. (2024); mushrooms, serum, urine, simulated gastric fluid	Multi-matrix validation study	Multi-class LC-MS/MS with matrix-dependent extraction/calibration	Broad multi-toxin screening: calibration 0.05-200 μg/L for the multi-analyte panel; reported recovery 73.0-110.3% and RSD <=19.4%.	Direct analytical evidence	Performance varies by analyte and matrix; broad-screening validation is not identical to an IA/MUS-specific mushroom method.	6.3, 6.5
[55]	Katragunta et al. (2025); consumer mushroom products	27 products: 14 gummies, 3 chocolates, 6 capsules, 1 tablet, 3 powders	Validated quantitative LC-QToF-MS targeting IA, MUS, muscarine, psilocin, and psilocybin	Demonstrated substantial label-composition variability; among 11 gummies claiming Amanita extracts, MUS and muscarine were detected while IA was not detected.	Direct product evidence	Convenience sample of 27 products; five-target panel; cannot support market-wide prevalence estimates.	6.7, 9.1-9.6
[59]	Dai et al. (2025); mice exposed to ibotenic acid	Animal experiment	Time-course neurochemical measurements after IA exposure	Provides experimental evidence of IA-associated changes in brain neurotransmitter systems.	Direct for the mouse model; indirect for human poisoning	Animal dosing/model does not establish human toxic dose or clinical kinetics.	7.1, 7.5
[60]	Moss and Hendrickson (2019); poison-centre reports	Regional poison-centre data, 2002-2016	Retrospective clinical/toxicological surveillance	Characterizes reported toxicity from muscimol/ibotenic-acid-containing mushrooms in clinical practice.	Direct observational clinical evidence	Species/exposure confirmation and chemical quantification are incomplete in many poison-centre cases.	7, 9
[61]	Tamminga, Crayton, and Chase (1978); schizophrenia	6 patients	Controlled administration of purified muscimol	Provides historical direct human pharmacology and tolerability observations for purified muscimol.	Direct human pharmacological evidence	Very small selected sample; historical therapeutic dosing is not a safety threshold for mushroom exposure.	7.5
[62]	Shoulson et al. (1978); Huntington disease	10 patients	Double-blind administration of purified muscimol	Provides historical human CNS pharmacology data for oral muscimol.	Direct human pharmacological evidence	Small selected clinical sample; not an intoxication or dose-threshold study.	7.5
[63]	Tamminga et al. (1978); human neuroendocrine response to muscimol	9 subjects; study-specific oral doses in the 5-9 mg range	Controlled human administration with prolactin and growth-hormone endpoints	Demonstrates direct human pharmacodynamic effects of purified muscimol.	Direct human pharmacological evidence	Specialized endocrine endpoints and selected clinical groups; cannot define toxic dose in Amanita exposure.	7.5
[64]	Tamminga, Crayton, and Chase (1979); tardive dyskinesia	8 neuroleptic-free subjects; oral 5-9 mg muscimol	Clinical pharmacology	Historical evidence that purified muscimol produces measurable CNS effects in humans at milligram doses and attenuated involuntary movements in this selected group.	Direct human pharmacological evidence	Small historical therapeutic study; not a poisoning safety study.	7.5
[65]	Vendramin and Brvar (2014); A. muscaria / A. pantherina poisoning	Clinical series / syndrome comparison	Clinical toxicology	Supports distinction and overlap of the two isoxazole-mushroom poisoning syndromes.	Direct clinical evidence for described cases	Clinical identification and ingested chemical dose are not uniformly analytically confirmed.	7
[66]	Ebbens et al. (2024); Diamond Shruumz product-associated neurotoxicity	1 case	Case report	Documents clinically important neurotoxicity associated with a marketed mushroom product.	Direct clinical product-exposure evidence	Single case; complex product composition and possible co-exposures limit attribution to IA/MUS.	7, 9
[67]	Aigumov et al. (2025); fungal samples and biological matrices	Method-development study	HPLC-MS/MS for muscarine, MUS, and IA	Extends modern tandem-MS detection across fungal and biological matrices.	Direct analytical evidence	Matrix-transferability and validation performance remain application-specific.	6

Ref.	Study / species or matrix	Study size	Analytical / experimental approach	Principal finding / claim supported	Evidence category	Main limitation	Review section(s)
[68]	Stępień-Pyśniak et al. (2026); Old Polish ducks	Veterinary fatal-poisoning report	Veterinary toxicology and diagnostic work-up	Demonstrates that Amanita-associated isoxazole poisoning can be fatal in avian veterinary exposure.	Direct veterinary evidence	Non-human species; cannot be extrapolated directly to human dose-response.	7.8
[69]	Romano et al. (2019); dog, PCR-confirmed A. muscaria	1 dog	Veterinary case report plus PCR identification of mushroom material	Provides species-confirmed evidence of fatal A. muscaria poisoning in a dog.	Direct veterinary evidence	Single non-human case; limited chemical dose information.	7.8
[70]	Li et al. (2022); Cenococcum geophilum under drought stress	Experimental fungal transcriptomics study	Transcriptomic response to drought in another ectomycorrhizal fungus	Shows that drought can alter ectomycorrhizal fungal physiology and gene expression, providing mechanistic context for environmental hypotheses.	Indirect / hypothesis-generating for A. muscaria IA/MUS	Different fungal species; no IA/MUS pathway measurement.	8, 10.4
[71]	Falandysz, Mędyk, and Treu (2018); A. muscaria from northern Poland	A. muscaria field material	Elemental analysis	Demonstrates bioconcentration and associations of heavy metals in A. muscaria.	Direct for element accumulation; indirect for IA/MUS regulation	No direct measurement linking element accumulation to isoxazole biosynthesis.	8
[72]	Hanć et al. (2021); developing and mature A. muscaria	Developmental fruiting bodies	Mercury and selenium analysis	Shows developmental variation in selected elements in A. muscaria.	Direct for elemental data; indirect for IA/MUS regulation	Elemental variation is not evidence of altered ibotenic-acid pathway activity.	8
[73]	Dunk et al. (2012); A. muscaria ectomycorrhiza with Nothofagus	Ectomycorrhizal association study	Mycorrhizal characterization	Provides direct ecological evidence for A. muscaria-host association and resource-exchange context.	Direct ecological context; hypothesis-generating for IA/MUS regulation	No IA/MUS measurements or pathway-regulation endpoint.	8
[74]	Ordak et al. (2023); A. muscaria consumers	Survey study	Self-reported reasons, forms of ingestion, and adverse effects	Provides direct evidence of contemporary use patterns and self-reported effects.	Direct for reported behaviour; indirect for chemically verified exposure	Self-reported mushroom/product identity and dose; no standardized chemical verification.	7, 9

IA, ibotenic acid; MUS, muscimol; LC-MS/MS, liquid chromatography-tandem mass spectrometry; UPLC-MS/MS, ultra-performance liquid chromatography-tandem mass spectrometry; UHPLC-MS/MS, ultra-high-performance liquid chromatography-tandem mass spectrometry; NR, not reported in a directly comparable form. Methodological standards, narrative reviews, and general fungal-mechanism sources used only for background or validation guidance are not listed as independent IA/MUS datasets in this table.

Table S4. Commercial-product evidence relevant to composition, labelling, and analytical interpretation of products marketed as containing Amanita muscaria or other mushroom nootropics.

Trade names are reproduced solely to identify the products tested in the cited source reports and do not imply endorsement. Product-level findings are time- and location-specific and should not be interpreted as estimates of market prevalence.

Study / product	Labelled or marketed ingredients / claim	Compounds detected in the reported analysis	Method	Quantitative result	Principal limitation / interpretation
[28] Diamond Shruumz Sour Peach Apple	Mushroom nootropic; caffeine; lion's mane; chaga; reishi	Psilocin	Qualitative LC-QToF-MS library matching	NR (qualitative screen)	IA, MUS, and muscarine were not included in the matching library; their presence was undetermined. A qualitative negative result for a compound not in the library cannot establish absence.
[28] Diamond Shruumz Rainbow	Mushroom nootropic; caffeine; lion's mane; chaga; reishi	Psilocin; caffeine	Qualitative LC-QToF-MS library matching	NR (qualitative screen)	Same library limitation as above; one of six packages in a local retail snapshot.
[28] Urb Magic Amanita Mushroom Watermelon	Label included muscimol, ibotenic acid, muscarine, and A. muscaria extracts	Psilocybin; psilocin; hordenine; 2-phenethylamine	Qualitative LC-QToF-MS library matching	NR (qualitative screen)	Detected compounds did not correspond simply to the Amanita-focused label claims. IA/MUS/muscarine could not be evaluated with the reported library.
[28] Wonderland Legal Psychedelics Cherry Nirvana	A. muscaria extract; blue lotus; reishi; lion's mane; cordyceps	Psilocin; N,N-DMT; caffeine; mitragynine	Qualitative LC-QToF-MS library matching	NR (qualitative screen)	Complex multi-ingredient product; qualitative result cannot quantify dose. IA/MUS/muscarine were not in the matching library.
[28] Psilly's Legal Psychedelic Mushrooms Fruit Punch	Fly agaric (A. muscaria) extract plus hemp-derived extract	Ephedrine	Qualitative LC-QToF-MS library matching	NR (qualitative screen)	A single retail package cannot characterize the brand or market; Amanita isoxazoles were not assessable with the source library.
[28] Tryp mushroom gummies	Lion's mane; reishi; cordyceps; maitake; turkey tail; hemp extract	None identified by the reported screen	Qualitative LC-QToF-MS library matching	NR (qualitative screen)	No compound match in this targeted/library-based qualitative analysis does not imply complete chemical characterization.
[28] Michienzi et al. retail survey, overall	Six packages representing five brands purchased near Charlottesville, Virginia, October 2023	Several undeclared psychoactive or other compounds identified across products	Qualitative LC-QToF-MS	6 packages / 5 brands; not a quantitative concentration study	Local, convenience snapshot. IA, MUS, and muscarine were absent from the matching library and therefore remained undetermined.
[55] Katragunta et al. consumer-product dataset	27 products: 14 gummies, 3 chocolates, 6 capsules, 1 tablet, and 3 powders; 11 of 14 gummies claimed Amanita mushroom extracts	Among the 11 Amanita-claim gummies, MUS and muscarine were detected while IA was not detected. Across all 27 products, 11 contained psilocin plus psilocybin; five contained none of the five target analytes; one product contradicted a "contains no psilocybin" claim.	Validated quantitative LC-QToF-MS targeting IA, MUS, muscarine, psilocin, and psilocybin	Product-specific concentrations varied; aggregate detection findings are summarized here. Authentic A. muscaria reference-sample concentrations reported in the study are not consumer-product concentrations.	Twenty-seven-product convenience sample and five-target panel; informative for label-composition discordance but not representative of prevalence across the wider market.

LC-QToF-MS, liquid chromatography-quadrupole time-of-flight mass spectrometry; IA, ibotenic acid; MUS, muscimol; NR, not reported as a quantitative concentration. The main manuscript treats products of uncertain fungal identity as commercial exposure matrices rather than authenticated A. muscaria specimens.
